# Disease modeling of a mutation in α‐actinin 2 guides clinical therapy in hypertrophic cardiomyopathy

**DOI:** 10.15252/emmm.201911115

**Published:** 2019-11-03

**Authors:** Maksymilian Prondzynski, Marc D Lemoine, Antonia TL Zech, András Horváth, Vittoria Di Mauro, Jussi T Koivumäki, Nico Kresin, Josefine Busch, Tobias Krause, Elisabeth Krämer, Saskia Schlossarek, Michael Spohn, Felix W Friedrich, Julia Münch, Sandra D Laufer, Charles Redwood, Alexander E Volk, Arne Hansen, Giulia Mearini, Daniele Catalucci, Christian Meyer, Torsten Christ, Monica Patten, Thomas Eschenhagen, Lucie Carrier

**Affiliations:** ^1^ Institute of Experimental Pharmacology and Toxicology University Medical Center Hamburg‐Eppendorf Hamburg Germany; ^2^ DZHK (German Centre for Cardiovascular Research), partner site Hamburg/Kiel/Lübeck Hamburg Germany; ^3^ Department of Cardiology‐Electrophysiology University Heart and Vascular Center Hamburg Germany; ^4^ Institute of Genetics and Biomedical Research Milan Unit National Research Council Milan Italy; ^5^ Humanitas Clinical and Research Center Rozzano, Milan Italy; ^6^ Faculty of Medicine and Health Technology Tampere University Tampere Finland; ^7^ Bioinformatics Core University Medical Center Hamburg‐Eppendorf Hamburg Germany; ^8^ Department of General and Interventional Cardiology University Heart and Vascular Center Hamburg Germany; ^9^ Radcliffe Department of Medicine University of Oxford Oxford UK; ^10^ Institute of Human Genetics University Medical Center Hamburg‐Eppendorf Hamburg Germany; ^11^ Present address: Department of Cardiology Boston Children's Hospital Harvard Medical School Boston MA USA

**Keywords:** disease modeling, human‐induced pluripotent stem cells, hypertrophic cardiomyopathy, long QT syndrome, precision medicine, Cardiovascular System, Genetics, Gene Therapy & Genetic Disease, Regenerative Medicine

## Abstract

Hypertrophic cardiomyopathy (HCM) is a cardiac genetic disease accompanied by structural and contractile alterations. We identified a rare c.740C>T (p.T247M) mutation in *ACTN2*, encoding α‐actinin 2 in a HCM patient, who presented with left ventricular hypertrophy, outflow tract obstruction, and atrial fibrillation. We generated patient‐derived human‐induced pluripotent stem cells (hiPSCs) and show that hiPSC‐derived cardiomyocytes and engineered heart tissues recapitulated several hallmarks of HCM, such as hypertrophy, myofibrillar disarray, hypercontractility, impaired relaxation, and higher myofilament Ca^2+^ sensitivity, and also prolonged action potential duration and enhanced L‐type Ca^2+^ current. The L‐type Ca^2+^ channel blocker diltiazem reduced force amplitude, relaxation, and action potential duration to a greater extent in HCM than in isogenic control. We translated our findings to patient care and showed that diltiazem application ameliorated the prolonged QTc interval in HCM‐affected son and sister of the index patient. These data provide evidence for this *ACTN2* mutation to be disease‐causing in cardiomyocytes, guiding clinical therapy in this HCM family. This study may serve as a proof‐of‐principle for the use of hiPSC for personalized treatment of cardiomyopathies.

## Graphical abstract

Disease modeling of a rare ACTN2 mutation in iPSC‐derived cardiomyocytes & heart tissues engineering revealed typical features of hypertrophic cardiomyopathy & electrophysiological anomalies. Diltiazem reversed the *in vitro* phenotypes & guided clinical therapy in the family, reducing QTc intervals.

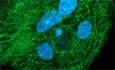

## Introduction

Hypertrophic cardiomyopathy (HCM) is an inherited myocardial disease with a prevalence of 1:500 in the general population (Maron *et al*, 1995). It is defined by hypertrophy, mainly seen in the left ventricle (LV) and associated with myocardial disarray, fibrosis, and diastolic dysfunction (Ho, 2010). Patients with HCM exhibit frequent episodes of atrial fibrillation (22%), non‐sustained ventricular tachycardia (31%), and annual incidence of sudden cardiac death (1%) (Adabag *et al*, 2005).

There is no curative treatment for HCM, which reverses or prevents hypertrophy and dysfunction of the heart (Tardiff *et al*, 2015). Available drug‐based therapies aim at relieving HCM‐associated symptoms and decelerating disease progression. β‐adrenoceptor antagonists (β‐blockers) and Ca^2+^‐channel blockers are used to reduce heart rate and therefore lengthen LV filling time (Marian, 2009). While these therapeutic approaches address general disease mechanisms independent of the underlying pathophysiology, knowledge of the disease‐causing mutation(s) and related cardiomyocyte (CM)/heart function could theoretically inform about specific individual treatment options, the concept of personalized medicine.

Over 1,500 mutations in at least 11 different genes, encoding components of the cardiac sarcomere, have been identified as a potential cause for HCM (Marian & Braunwald, 2017), highlighting the need for precision medicine as genetic diversity of HCM‐affected individuals is paralleled by clinical heterogeneity. Furthermore, the causative role of many gene mutations remains controversial in HCM, one example being *ACTN2*, which encodes α‐actinin 2. The α‐actinin 2 protein is located at the Z‐disk of the sarcomere in homodimers fulfilling three main functions: sarcomere formation, anchoring/crosslinking of actin thin filaments, and interaction with titin (Gautel & Djinovic‐Carugo, 2016; Chopra *et al*, 2018). Several *ACTN2* variants have been associated with HCM, but only a few could be validated as disease‐causing with altered function (Theis *et al*, 2006; Chiu *et al*, 2010; Girolami *et al*, 2014; Haywood *et al*, 2016). One of these studies revealed reduced F‐actin‐binding affinity, altered Z‐disk localization and dynamic behavior of two α‐actinin 2 mutants after gene transfer in CMs (Haywood *et al*, 2016).

Here, we identified a novel *ACTN2* mutation (c.740C>T; p.T247M) in a patient with HCM and set out to study its consequences on cellular function, using human‐induced pluripotent stem cell (hiPSC)‐derived CMs and CRISPR/Cas9 gene editing, an approach increasingly employed to study disease mechanisms in cardiomyopathies (Prondzynski *et al*, 2017; Mosqueira *et al*, 2018; Eschenhagen & Carrier, 2019). We also used engineered heart tissues (EHTs) to study contractile and electrophysiological function in a more physiological and mature 3D format (Uzun *et al*, 2016; Lemoine *et al*, 2017, 2018).

## Results

### Identification of a novel *ACTN2* mutation in an HCM proband and family

**Figure 1:**
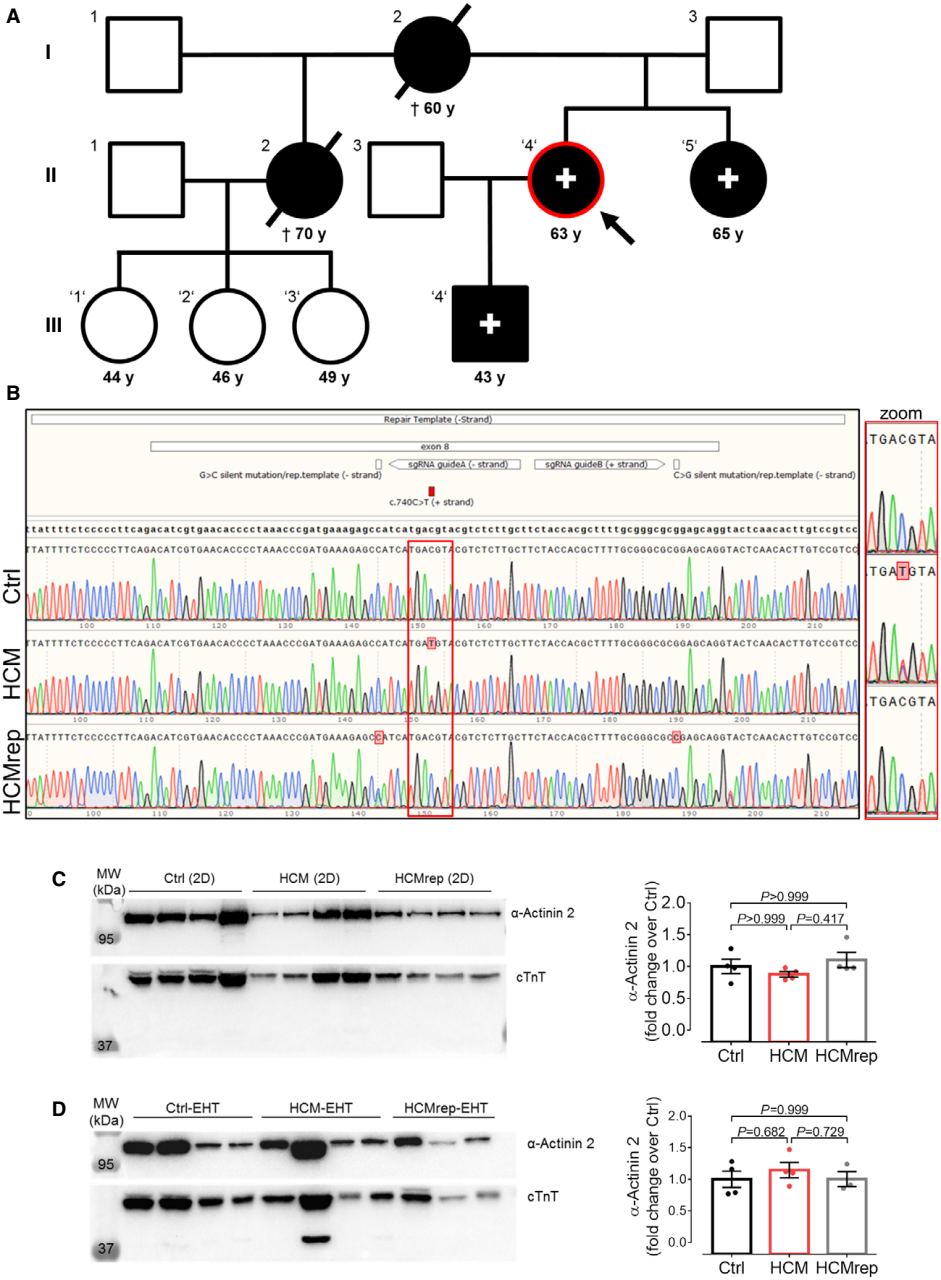
HCM‐affected family and molecular characterization of human tissue and hiPSC‐derived CMs from 2D and 3D models APedigree of HCM‐affected family carrying a novel *ACTN2* mutation (c.740C>T; T247M). Individuals who were genotyped are marked with an apostrophe, and HCM patients are indicated by filled symbols and carriers of this *ACTN2* mutation with a (+) sign. Encircled in red with the arrow is the index patient (II.4) from whom the HCM hiPSC line was created.BRepresentative Sanger sequencing results of the *ACTN2* locus in the Ctrl, HCM, and HCMrep hiPSCs (higher magnification of the locus shown on the right, red rectangle). Depicted is the mutation on position g.54,208 (c.740C>T), the repair template from g.54,148 to 54,270 (‐strand), the according silent mutations encoded on the repair template on position g.54,200 (G>C; ‐strand) and g.54,245 (C>G; ‐strand), and sgRNAs A and B.C, DWestern blot analysis of hiPSC‐CMs (C) and ‐EHTs (D) stained with antibodies directed against α‐actinin 2 and cTnT. Quantification of α‐actinin 2 protein levels in single samples, normalized to cTnT and related to Ctrl, is depicted [*n* = number of samples/differentiations; 2D: Ctrl (*n* = 4/3); HCM (*n* = 4/3); HCMrep (*n* = 4/2); EHT: Ctrl (*n* = 4/2); HCM (*n* = 4/3); HCMrep (*n* = 3/3)]. Data are expressed as mean ± SEM, one‐way ANOVA with Bonferroni's post‐test. Abbreviations used are as follows: CMs, cardiomyocytes; cTnT, cardiac troponin T; Ctrl, control; EHTs, engineered heart tissues; MW, molecular weight marker. Source data are available online for this figure.

DNA sequencing of 16 HCM‐associated genes (Appendix Table S1) revealed an *ACTN2* c.740C>T (p.T247M) variant in a HCM patient (II.4; Fig 1A), who presented with LV hypertrophy, LV outflow tract (LVOT) obstruction, atypical atrial flutter, and paroxysmal atrial fibrillation. This variant is not listed in the genome aggregation database (gnomAD), and different *in silico* prediction tools (CADD, REVEL, M‐CAP) revealed high pathogenicity scores. The HCM‐affected son (III.4) and sister (II.5) of the index patient carried this *ACTN2* mutation, but not the nieces (III.1, III.2, III.3; Fig 1A).

The index patient II.4 underwent myectomy at the age of 58, a surgical ablation and mitral valve repair at 59 years, and a mitral valve replacement at age 64. After myectomy, she developed a left bundle branch block. Atypical flutter was ablated when she was 65 years old. Patient II.5 suffered from paroxysmal supraventricular tachycardia since adolescence. At the age of 56 years, she was diagnosed with HCM, and an electrophysiological examination revealed an atrioventricular nodal reentrant tachycardia, which was treated by slow pathway modulation. She underwent pulmonary vein isolation for paroxysmal atrial fibrillation at the age of 59 and 61 years. Patient III.4 was diagnosed with HCM at the age of 43 in the course of this study. Long‐term ECG analysis revealed a low number of premature atrial contractions and polymorphic premature ventricular contractions as single events and as couplets. Cardiac magnetic resonance imaging showed a normal left ventricular function, a moderate septal hypertrophy of 14 mm with an apical pronunciation and without LVOT obstruction. Late gadolinium enhancement appeared in the anterior septum at the insertion to the right ventricle. The half‐sister (II.2) of the index patient was diagnosed with HCM at the age of ~55 years with LVOT obstruction. She underwent transcoronary ablation of septum hypertrophy at the age of 59 years, developed supraventricular premature beats, paroxysmal atrial fibrillation, apoplexy, and died at the age of 70 years after post‐operative sepsis more than 10 years ago in an external clinic before the investigation of this study. Interestingly, supraventricular premature beats were reported to be reduced by verapamil, but not by bisoprolol. Clinical characterization of affected versus non‐affected family members is shown in Table 1. In this HCM family, there is no hint for an early manifestation of HCM and none of the family members reported an early unexpected sudden death in the family.

**Table 1:** Clinical characteristics of members of the HCM‐affected family

	Ctrl (*n* = 3)	HCM (*n* = 3)	*P* value
	Mean ± SEM	Mean ± SEM	
Age (years)	46.3 ± 1.8	57.0 ± 8.6	0.211
Body surface (m²)	2.0 ± 0.1	2.1 ± 0.3	0.769
Heart rate (bpm)	84 ± 15	76 ± 5	0.577
QT (ms)	359 ± 19	406 ± 17	0.089
QTcB (ms)	420 ± 13	458 ± 8	0.023
QTcF (ms)	398 ± 1	440 ± 8	0.003
SBP (ms)	139 ± 4	151 ± 7	0.142
DBP (ms)	85 ± 5	82 ± 9	0.709
LVEDD (mm)	39 ± 2	45 ± 4	0.125
LVESD (mm)	27 ± 3	36 ± 3	0.056
LVEDV (ml)	63 ± 6	94 ± 26	0.236
LVESV (ml)	20 ± 1	38 ± 13	0.180
LVWTmax (mm)	13.1 ± 0.6	17.6 ± 0.1	0.001
LVEF (%)	63 ± 1	61 ± 4	0.535
Global E′ (cm/s)	12.9 ± 0.9	8.1 ± 1.4	0.024
E (cm/s)	83 ± 8	105 ± 27	0.384
A (cm/s)	75 ± 15	42 ± 4	0.067
E/A	1.1 ± 0.1	2.5 ± 0.5	0.025
E/E′	6.5 ± 0.2	14.3 ± 6.1	0.192
Deceleration time (ms)	116 ± 6	206 ± 22	0.007
LA vol (ml)	42 ± 3	71 ± 5	0.006

Three family members as controls (Ctrl), negative (III.1/2/3) for *ACTN2* mutation (c.740C>T; T247M), compared to three family members positive for *ACTN2* mutation (HCM; II‐4, II‐5, III‐4). Data are expressed as mean ± SEM. *P* values derived by unpaired Student's *t*‐test; abbreviations used are as follows: QTcB, Bazett‐corrected QT; QTcF, Fredericia‐corrected QT; SBP, systolic blood pressure; DBP, diastolic blood pressure; LVEDD, left ventricular end‐diastolic diameter; LVESD, LV end‐systolic diameter; LVEDV, LVED volume; LVESV, LVES volume; LVWTmax, maximum LV wall thickness; LVEF, LV ejection fraction; LA, left atrium; Global E′, early myocardial tissue Doppler relaxation velocity.

### Patient‐derived hiPSC‐CMs exhibit the same level of α‐actinin 2 than CRISPR/Cas9‐generated control

In order to characterize this rare *ACTN2* variant *in vitro*, dermal fibroblasts from the index patient (II.4) were reprogrammed to hiPSCs (HCM) and used to generate the isogenic control cell line (HCMrep) with the CRISPR/Cas9 nickase approach and homology‐directed repair with an exogenous template containing the WT *ACTN2* sequence (Fig 1B, Appendix Fig S1A). Both cell lines were compared to hiPSC derived from a donor individual (Ctrl). As none of the hiPSC lines presented karyotype abnormalities (Appendix Fig S1B), they were differentiated to CMs (Breckwoldt *et al*, 2017) and subjected to our disease modeling protocol (Appendix Fig S1C). Genotypes were confirmed in genomic DNA from hiPSCs (Fig 1B) and hiPSC‐CMs (data not shown).

We then analyzed the α‐actinin 2 amount in hiPSC‐CMs cultured in 2D and EHT format as well as in LV septum from the index patient II.4. α‐Actinin 2 protein levels in hiPSC‐CMs did not differ either between lines and culture formats (Fig 1C and D) or between patient II.4 septal myectomy and non‐failing heart tissue (Appendix Fig S2A).

To evaluate allelic expression of wild‐type (WT) and mutant alleles, we subcloned and sequenced RT–PCR fragments from HCM‐EHTs and from the myectomy tissue sample of patient II.4. Analysis of ≥ 13 clones each revealed 48 and 60% mutant clones in EHT and LV septum of the patient, respectively (Appendix Fig S2B), suggesting allelic balance and stable missense transcripts. Allelic balance was confirmed by RNA sequencing (Appendix Fig S2C). Total transcript levels of *ACTN2* were ~two‐fold higher in all HCM samples (hiPSC in 2D, 3D, and human myectomy) than in their corresponding controls (Appendix Fig S2D). Genes related to hypertrophic signaling (*MYH7, NPPB, SRF, FHL1*), Ca^2+^ handling (*CACNA1C, PLN, RYR2*), fibrosis (*COL1A1, CTGF*), and apoptosis (*BCL2*) were dysregulated in HCM myectomy and in HCM hiPSC‐CMs or ‐EHTs (Appendix Fig S2D).

### Patient‐derived hiPSC‐CMs exhibit HCM phenotypes

**Figure 2:**
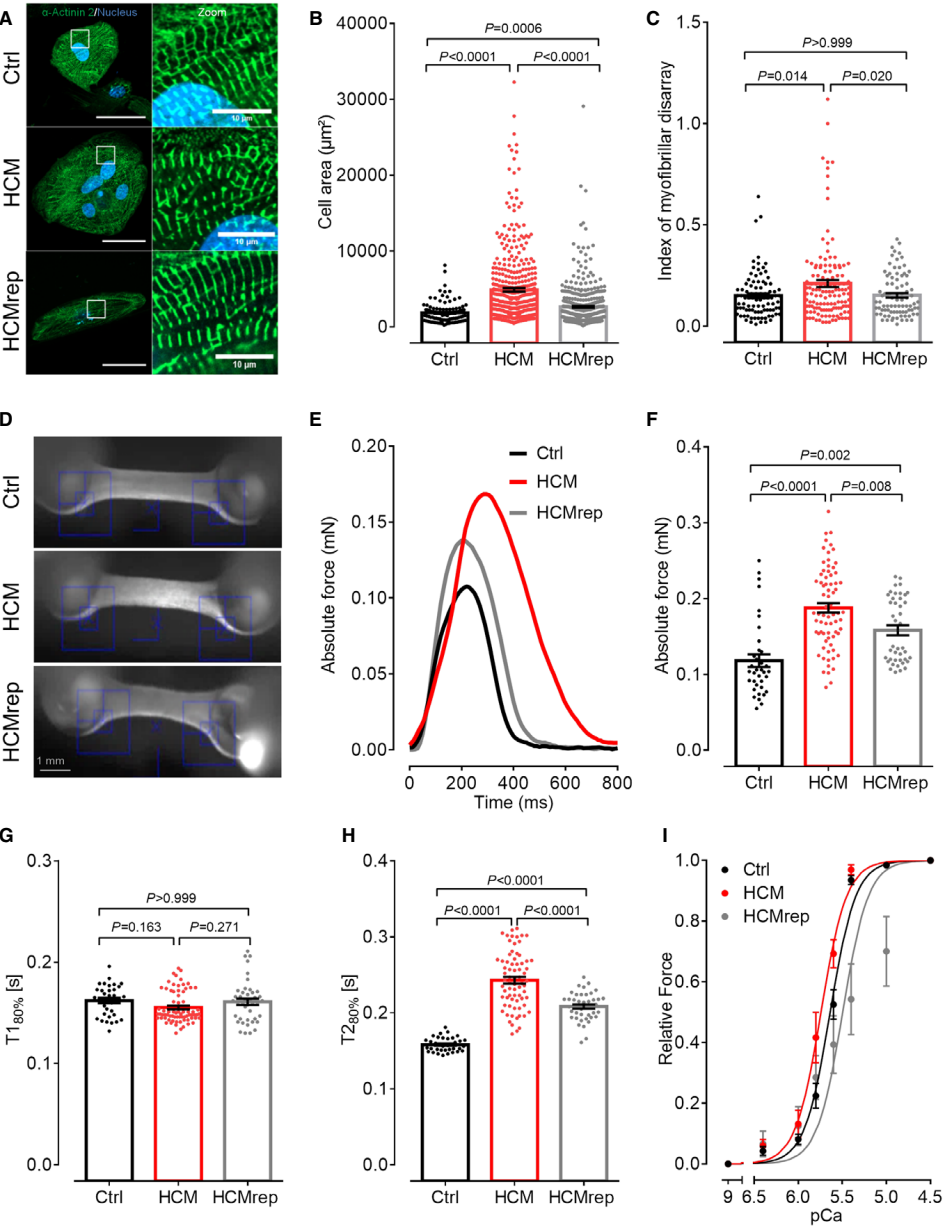
Disease modeling with 2D‐ and 3D‐cultured hiPSC‐derived CMs ARepresentative immunofluorescence images of the Ctrl, the HCM, and isogenic control HCMrep. HiPSC‐CMs were stained after 30 days in 2D culture with an antibody against α‐actinin 2 and Hoechst for nuclei staining (scale: 50 μm) and with higher magnification (scale: 10 μm).BQuantification of cell area analyzed with Fiji software [*n* = number of analyzed cells/wells/differentiations: Ctrl (*n* = 112/11/3); HCM (*n* = 424/7/4); HCMrep (*n* = 443/5/3)].CBlinded analysis of myofibrillar disarray using high‐resolution pictures [*n* = number of analyzed sarcomeres/cells/differentiations: Ctrl (*n* = 90/30/3); HCM (*n* = 132/44/2); HCMrep (*n* = 86/29/2)].DRepresentative EHTs from each cell line.ERepresentative single traces of EHTs in 1.8 mM Ca^2+^ Tyrode's solution under 1‐Hz pacing at 37°C.F–HFunctional parameters of force (F), time to peak 80% (T1_80%_, G), and time from peak to baseline 80% (T2_80%_, H) were measured under paced conditions at 1 Hz in 1.8 mM Ca^2+^ Tyrode's solution at 37°C [*n* = number of measurements/EHTs/differentiations: Ctrl (*n* = 36/25/4); HCM (*n* = 64/60/6); HCMrep (*n* = 41/29/4)].IForce–pCa relationships were performed with the Aurora 1400A system in skinned EHT muscle strips from respective cell lines [*n* = number of measured and skinned EHT strips/number of EHTs/number of batches: Ctrl (*n* = 14/7/3); HCM (*n* = 10/4/2); HCMrep (*n* = 7/7/2)]. Concentration–response curves were fitted to the data points, and force–pCa relationship comparison was done by using extra sum‐of‐squares *F*‐test.Data information: Data are expressed as mean ± SEM, one‐way ANOVA with Bonferroni's post‐test.

To further evaluate morphological impacts of this novel *ACTN2* mutation, hiPSC‐CM lines were seeded at low‐density in 96‐well plates (Appendix Fig S1C). Immunofluorescence analysis of hiPSC‐CMs with an α‐actinin 2 antibody showed a cross‐striated pattern at 30 days *in vitro* (Fig 2A), indicating proper formation of sarcomeres. Cell area was markedly higher in hiPSC‐CMs from HCM than from Ctrl and HCMrep (Fig 2B). Analysis of Z‐disk structure showed a higher index of myofibrillar disarray in HCM than in Ctrl and HCMrep (Fig 2C).

To explore functional consequences of the mutation, we casted hiPSC‐CM in 3D format as EHTs and analyzed their contractile behavior after 26 days *in vitro* (Fig 2D). Contraction traces of EHTs paced at 1 Hz showed 58 and 19% higher peak force in HCM‐ than in Ctrl‐ and HCMrep‐EHTs, respectively (Fig 2E and F). Time to peak contraction (T1_80%_) did not differ between experimental groups (Fig 2G). Relaxation time (T2_80%_) was 54 and 17% longer in HCM‐ than in Ctrl‐ and HCMrep‐EHTs, respectively (Fig 2E and H). These data suggest increased contractility and relaxation deficit as an underlying disease phenotype in HCM‐EHTs.

To evaluate whether the prolonged relaxation in HCM‐EHTs is due to higher myofilament Ca^2+^ sensitivity, a common finding in HCM, we chemically skinned EHTs and human tissue samples and evaluated the force–pCa relationships. In line with the higher peak force in intact HCM‐EHTs (Fig 2F), force normalized to cross‐sectional area was higher in skinned HCM‐EHTs than in Ctrl‐ or HCMrep‐EHTs (Appendix Fig S3A), whereas it did not differ between myectomy of patient II.4 and non‐failing heart sample (Appendix Fig S3B). The force–Ca^2+^ relationship was shifted to the left in skinned HCM‐EHTs compared to HCMrep and Ctrl‐EHTs (Fig 2I; Appendix Fig S3C). Similarly, pCa_50_ was higher in skinned HCM than in non‐failing heart muscle strips (Appendix Fig S3D and E). These data suggest that increased myofilament Ca^2+^ sensitivity could contribute to the prolonged relaxation in HCM‐EHTs.

### HCM hiPSC‐CMs exhibit longer action potential, higher L‐type Ca^2+^ channel current density, and reduced binding of α‐actinin 2 to L‐type Ca^2+^ channels

**Figure 3:**
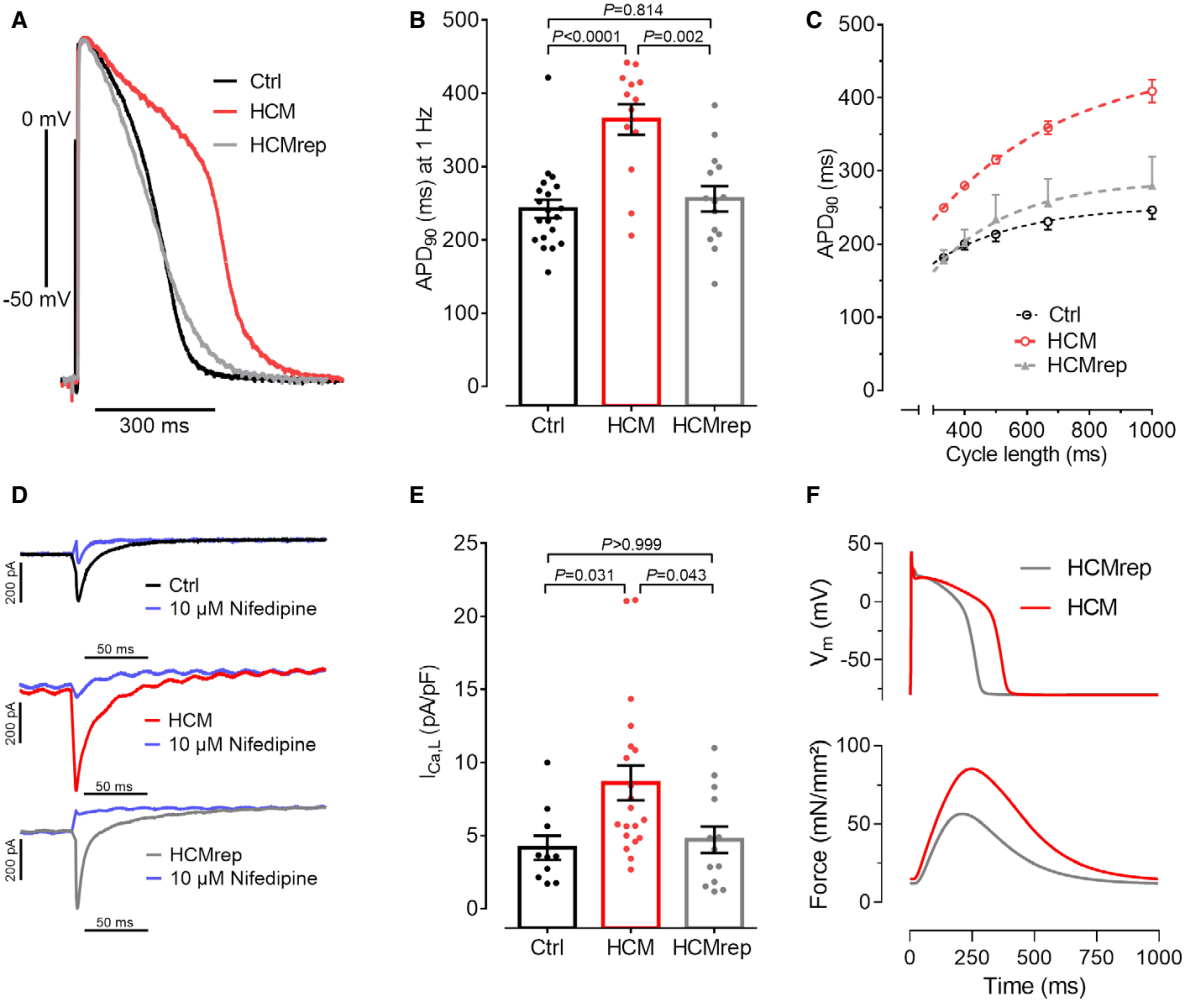
Electrophysiological characterization with 3D‐ and 2D‐cultured hiPSC‐derived CMs ARepresentative action potentials (APs) measured with sharp microelectrode in EHTs at 1‐Hz pacing.BQuantification of AP duration at 90% repolarization (APD_90_) of Ctrl, HCM, and HCMrep [*n* = number of EHTs/differentiations; Ctrl (20/8), HCM (13/6), HCMrep (14/5)].CCycle length dependence of APD_90_ of Ctrl (*n* = 7/3), HCM (*n* = 5/2), and HCMrep (*n* = 3/1).D, ERepresentative traces (D) and quantification (E) of single‐cell patch clamp recordings of L‐type calcium currents [*n* = number of isolated cardiomyocytes/differentiations; Ctrl (10/3), HCM (20/3), HCMrep (13/3)].FSimulated effect of 1.5‐fold I_Ca,L_ density in *in silico* model of human hiPSC‐CM AP and Force.Data information: Data are expressed as mean ± SEM, one‐way ANOVA with Bonferroni's post‐test.

Given that the difference in myofilament Ca^2+^ sensitivity was much smaller than the difference in relaxation time between HCM and control cell lines, we further explored electrophysiological consequences of the *ACTN2* mutation. Action potentials (APs) were recorded with sharp microelectrodes in intact EHTs. AP parameters were measured at 1‐Hz pacing. There was no difference in take‐off potential and upstroke velocity between EHTs from Ctrl, HCM, and HCMrep (Table 2), suggesting similar sodium current function. On the other hand, AP amplitude was higher, and AP duration at 50% (APD_50_) and 90% (APD_90_) of repolarization was ~50% longer in HCM‐EHTs than in Ctrl‐ and HCMrep‐EHTs (Fig 3A and B; Table 2). Longer APD in HCM was consistent over the whole range of physiologically relevant frequencies between 1 and 3 Hz (Fig 3C).

**Table 2:** Action potential parameters in 3D‐cultured hiPSC‐derived EHT

	Ctr (*n* = 20)	HCM (*n* = 13)	HCMrep (*n* = 14)	*P* values		
				Ctrl versus HCM	Ctrl versus HCMrep	HCM versus HCMrep
TOP (mV)	−76.1 ± 0.8	−78.9 ± 1.6	−75.7 ± 0.8	0.181	0.683	0.062
APA (mV)	100.9 ± 1.5	116.0 ± 2.7	106.2 ± 1.4	0.003	0.962	0.003
Vmax (V/s)	217.2 ± 16.7	243.7 ± 39.9	208.5 ± 19.1	0.730	0.965	0.623
APD_50_ (ms)	154.9 ± 12.9	298.9 ± 20.5	176.3 ± 14.7	< 0.001	0.585	< 0.001
APD_90_ (ms)	242.2 ± 12.6	364 ± 21.0	256.0 ± 17.4	< 0.001	0.814	< 0.001

Summary of results for take‐off potential (TOP), action potential amplitude (APA), maximum upstroke velocity (V_max_), AP duration at 50 and 90% repolarization (APD_50/90_; *n* = number of EHTs). *P* values for multiple comparison with one‐way ANOVA and Bonferroni's post‐test.

Prolongation of APs might be explained by an increased L‐type Ca^2+^ channel (LTCC) current (I_Ca,L_) in HCM. Accordingly, we measured I_Ca,L_ by patch clamp technique in isolated hiPSC‐CM from a confluent 2D culture. The I_Ca,L_ density, expressed as nifedipine‐sensitive inward current, was higher in HCM than in Ctrl and HCMrep hiPSC‐CMs, respectively (Fig 3D and E). We used an established computer model for AP and force in hiPSC‐CM (Lemoine *et al*, 2018) in order to estimate whether the increased I_Ca,L_ could explain the observed APD prolongation. Indeed, increase of I_Ca,L_ by 50% showed similar APD prolongation, delayed relaxation, and enhanced force (Fig 3F), supporting the experimental data (Fig 2E and F). The observed increase in I_Ca,L_ might thus contribute to the electro‐mechanical phenotype in this *ACTN2*‐associated HCM.

It has been shown previously that α‐actinin 2 interacts with ion channels and contributes to their modulation, such as the LTCC complex (Maruoka *et al*, 2000; Lu *et al*, 2007; Eden *et al*, 2016; Hong & Shaw, 2017). In particular, by binding to the IQ segment of the Ca_v_ α1.2, pore unit of the LTCC, α‐actinin 2 modulates LTCC density and function at the plasma membrane (Sadeghi *et al*, 2002; Hall *et al*, 2013; Tseng *et al*, 2017). Since we found increased I_Ca,L_ in HCM hiPSC‐CMs and EHTs compared to Ctrl (Fig 3D and E), we tested whether the mutation in α‐actinin 2 might affect the interaction affinity between α‐actinin 2 and Ca_v_α1.2. By a bioluminescence resonance energy transfer (BRET) assay performed in live cardiac muscle‐like HL‐1 cells, we observed a similar binding affinity of Ca_v_α1.2 to WT and mutant α‐actinin 2 (Appendix Fig S4). However, while this binding affinity of Ca_v_α1.2 to WT α‐actinin 2 strongly increased with the co‐transfection of Ca_v_β2, the LTCC accessory subunit and chaperone of the LTCC pore unit, it did not in case of mutant α‐actinin 2. Thus, the HCM mutation decreased the interaction of α‐actinin 2 with the LTCC complex, possibly affecting the activity of the channel. This may explain the electro‐mechanical phenotype of the investigated HCM‐affected family, characterized by higher I_Ca,L_ density.

### Diltiazem intervention ameliorates the disease phenotype in HCM‐EHTs and the QT prolongation in HCM‐affected family members

**Figure 4:**
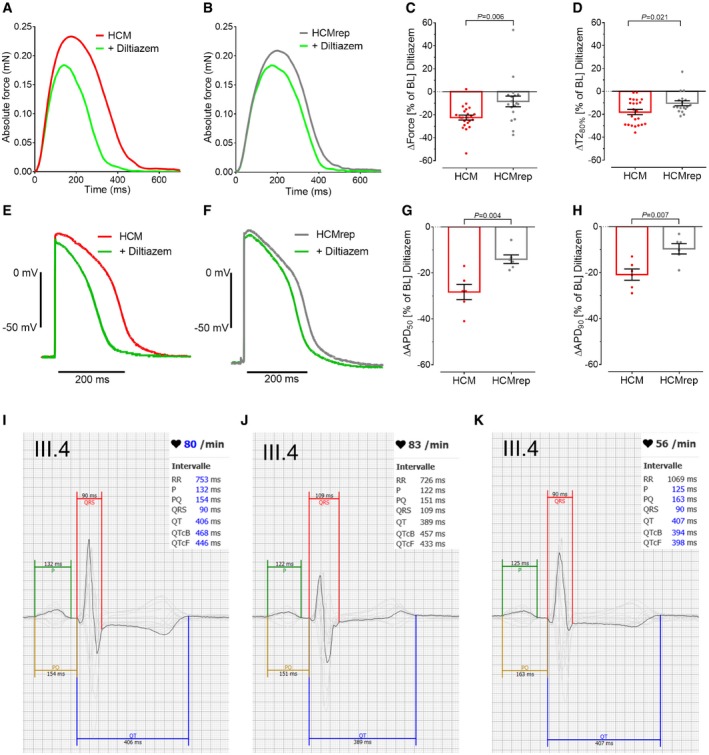
Diltiazem intervention in 3D‐cultured hiPSC‐derived EHT A, BRepresentative force traces at 1‐Hz pacing under baseline condition and with 3 μM diltiazem in HCM‐ (A) and HCMrep‐EHTs (B).C, DQuantification of the diltiazem effect in percent to baseline (BL) levels for force (C) and time to baseline 80% [T2_80%_, D; *n* = number of EHTs/differentiations; HCM (*n* = 23/2); HCMrep (*n* = 18/2)].E, FRepresentative action potentials (AP) paced at 1 Hz under BL condition (red for HCM, gray for HCMrep) and with 3 μM diltiazem (green) in HCM‐ (E) and HCMrep‐EHTs (F).G, HQuantification of AP duration at 50% (G) and 90% (H) of repolarization in HCM (*n* = 6/1) and HCMrep (*n* = 6/1).I–KRepresentative 12‐lead surface ECG with averaged signal and overlay of all leads (in gray, apart from V5 in black) of patient III.4 with different antihypertensive medication: (I) ramipril 5 mg, (J) ramipril 10 mg + 5 mg amlodipine, (K) ramipril 5 mg + diltiazem 180 mg for 1 month (QTcB: QT interval with Bazett correction; QTcF: with Fredericia correction).Data information: Data are expressed as mean ± SEM, unpaired Student's *t*‐test.

To follow the above reasoning, we tested whether the LTCC blocker diltiazem could reverse the contractile and electrophysiological phenotype of HCM‐EHTs. Treatment of EHTs with 3 μM diltiazem for 15 min reduced peak force to a significantly greater extent in HCM‐ (−29%; Fig 4A and C; Movies EV1 and EV2) than in HCMrep‐EHTs (−11%; Fig 4B and C; Movies EV3 and EV4). Similarly, diltiazem shortened relaxation to a greater extent in HCM‐ (−24.2%; Fig 4A and D) than in HCMrep‐EHTs (−11.7%; Fig 4B and D). In addition, diltiazem intervention led to a more pronounced AP shortening in HCM‐ (Fig 4E, G and H) than in HCMrep‐EHTs (Fig 4F, G and H). These results demonstrate that HCM‐EHTs are more susceptible to I_Ca,L_ block, supporting the hypothesis that increased I_Ca,L_ is an important contributor to the contractile and electrophysiological phenotype in this case of *ACTN2*‐associated HCM.

**Figure 5:**
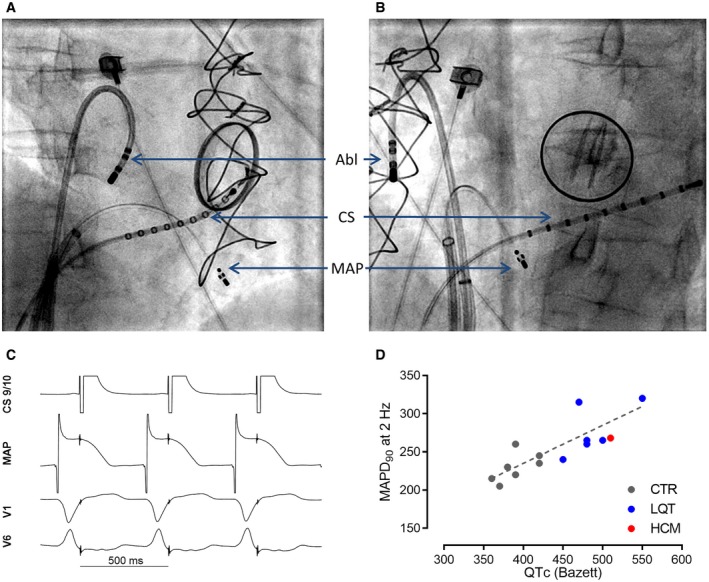
Monophasic action potential from HCM‐affected family member II.4 A, BFluoroscopy during the recording of monophasic action potentials in right anterior oblique projection (A) and left anterior oblique projection (B) at 30°. Atrial stimulation was performed by a catheter in coronary sinus (CS) and a second catheter for the recording of monophasic action potentials (MAPs) at the septum of the right ventricle, and the ablation catheter (Abl) was not used for the recording.CRepresentative MAPs traces.DComparison of MAPD_90_ and QTc (Bazett) in the index patients II.4 to a control and long QT (LQT) cohort taken from Shimizu *et al* (1991).

In line with this information, we found that corrected QT intervals (QT_c_) were in fact prolonged in affected versus non‐affected family members (Table 1, Appendix Fig S5). This information had not been given special consideration in clinical routine. Since patient II.4 underwent catheter ablation of atypical atrial flutter, we took the opportunity to record monophasic action potentials (MAPs) in the right ventricle (Fig 5A–C) as previously published (Shimizu *et al*, 1991). MAP duration at 90% of repolarization on the right ventricular septum was similar to a cohort with long QT (LQT) syndrome, but longer than in a control group as previously published (Fig 5D; Shimizu *et al*, 1991). In order to exclude a blended phenotype, we tested the index patient II.4 for mutations in the LQTS genes *CACNA1C*,* CALM1*,* CAV3*,* KCNE1*,* KCNE2*,* KCNH2*,* KCNJ2*,* KCNJ5*,* KCNQ1*,* SCN4B*,* SCN5A*, and *SNTA1* but could not identify a causative mutation (data not shown).

Based on our *in vitro* findings, we considered clinical implementation of diltiazem to ameliorate the electro‐mechanical phenotype of HCM‐affected family members. At this time, the index patient II.4 was still receiving antiarrhythmic treatment with amiodarone and bisoprolol because of recurrent atypical atrial flutter. Since we would not like to combine diltiazem with amiodarone and bisoprolol, we contacted patient III.4, who was receiving antihypertensive treatment with ramipril plus amlodipine, and patient II.5, who was receiving candesartan only. In case of patient III.4, we replaced amlodipine (a LTCC blocker with preferential vasodilatory effects) with diltiazem (a LTCC blocker with preferential cardiac action) (Lee & Tsien, 1983). At a standard dose (90 mg diltiazem, twice daily), QTc was reduced from 460 to 387 ms (Fig 4I–K). Reduced QTc was confirmed by 12‐lead ECGs over 24 h (averaged QTc ± 95% confidence interval): 446 ± 29 ms (5 mg ramipril, 5 mg amlodipine) to 418 ± 35 ms (5 mg ramipril, diltiazem 180 mg). In case of patient II.5, we added diltiazem (90 mg twice daily) and reduced candesartan from 8 to 4 mg. In consequence, QTc in 12‐lead ECG was reduced from 477 to 439 ms. These clinical data support our *in vitro* findings that increased I_Ca,L_ contributes to the electrophysiological phenotype and that its reduction by an LTCC blocker is efficient.

## Discussion

In this study, we applied hiPSCs and CRISPR/Cas9 gene editing to model a novel *ACTN2* missense mutation in an HCM‐affected family and showed that HCM hiPSC‐CMs recapitulated known hallmarks of HCM such as myofibrillar disarray, cell hypertrophy, increased myofilament Ca^2+^ sensitivity, hypercontractility, and prolonged relaxation. In addition, the *ACTN2* mutation resulted in AP prolongation and abnormal high I_Ca,L_ density. Given the availability of a clinically approved drug that reversed the contractile and electrophysiological phenotype *in vitro* (diltiazem), we could directly translate our findings to patient care and showed that diltiazem at standard dose reverted the LQT phenotype in the son and sister of the index patient. This study shows that patient‐specific hiPSC‐CMs can be used not only to describe disease‐related phenotypes, but also to individualize patient care for this specific *ACTN2* mutation.

The level of evidence for *ACTN2* being a causative gene in HCM is not strong as compared to *MYBPC3* and *MYH7* genes (Walsh *et al*, 2017). However, we provide several lines of evidence for this novel *ACTN2* mutation to be HCM‐causing: (i) It was segregated in three HCM‐affected members of the family, (ii) it was not found in the gnomAD browser, assembling > 130,000 exome or whole‐genome sequences from unrelated control individuals, and (iii) the patient‐derived hiPSC‐CMs developed a phenotype, which was corrected in the isogenic control. Our disease modeling approach revealed HCM phenotypes in 2D‐ and 3D‐cultured hiPSC‐CMs that were also reported previously in hiPSC‐CMs and animal models, such as myofibrillar disarray and CM hypertrophy (Lan *et al*, 2013; Dambrot *et al*, 2014; Tanaka *et al*, 2014; Ojala *et al*, 2016; Prondzynski *et al*, 2017), higher contraction force, longer relaxation time (Wijnker *et al*, 2016), and higher myofilament Ca^2+^ sensitivity (Fraysse *et al*, 2012; Eschenhagen & Carrier, 2019). The fact that all parameters were significantly different between the HCM and the isogenic control line carrying the same genetic background provides evidence for the mutation to be causative. However, significant differences in cell size, force, and relaxation time were also observed between HCMrep‐CMs and Ctrl‐CMs. Variability between control cell lines has been reported in several studies. For example, the response to diltiazem and other compounds varied between several control hiPSC‐CM lines (Lam *et al*, 2019). We also previously found significantly different APD90 in three different control hiPSC‐cardiomyocyte cell lines (Lemoine *et al*, 2018). More recently, we systematically analyzed in a blinded manner 36 control cell lines and found a standard deviation of the mean of 20–35% for several parameters of EHT contractile function (*Eschenhagen, personal communication*). Taken together, these studies underline the need of isogenic controls for personalized medicine.

The absence of a difference in α‐actinin 2 protein levels between HCM and control models combined with allelic balance of WT and mutant *ACTN2* mRNA transcripts suggests that the molecular mechanism of this *ACTN2* mutation is not haploinsufficiency but rather a gain‐of‐function mechanism with translation of mutant mRNA to an α‐actinin 2 protein with altered functional properties. We provide evidence for a defective Ca_v_β2‐dependent interaction of mutant α‐actinin 2 with the LTCC Ca_v_α1.2 pore‐forming unit, suggesting that WT α‐actinin 2 plays a relevant role in regulating LTCC assembly and function and that the channel activity is disturbed by the mutation. The physical interaction of α‐actinin 2 with Ca_v_α1.2 has previously been shown in human heart tissue by co‐immunoprecipitation (Lu *et al*, 2007) and is further dependent on the LTCC Ca_v_β2 auxiliary subunit as shown by our data. Since the HCM mutation is localized in the actin‐binding domain of α‐actinin 2 and not within the LTCC interacting region, we propose that the LTCC complex is correctly placed at the plasma membrane, but due to defective interaction with mutant α‐actinin 2, functional properties might be altered (Hall *et al*, 2013). Further studies will be dedicated to the unraveling of underlying mechanisms.

In order to translate *in vitro* findings to patient care, we evaluated how the electro‐mechanical phenotype could be pharmacologically modulated. HCM‐EHTs showed a significantly longer AP duration with a prolonged plateau phase, which could be caused by the higher I_Ca,L_ density. Indeed, diltiazem reduced force amplitude, relaxation, and APD to a greater extent in HCM‐ than in HCMrep‐EHTs. Similarly, restoration of diastolic function with diltiazem has been recently reported in hiPSC‐CMs carrying HCM mutations in *MYBPC3*,* MYH7*, or *TNNT2* (Wu *et al*, 2019). Prolonged APs in EHTs were in line with prolonged QTc intervals in all family members carrying the *ACTN2* mutation and with prolonged MAPs at the right ventricular septum of the index patient II.4. Therefore, we started treatment with diltiazem in patients III.4 and II.5 as an antihypertensive treatment. All clinically established LTCC blockers have both vasodilatory and cardiodepressant effects, only the ratio between the two principle actions differ (Eschenhagen, 2018). Diltiazem, in contrast to amlodipine, has relevant negative chronotropic, inotropic, and dromotropic effects at clinically used doses. The observation that the switch from amlodipine to diltiazem reduced the prolonged QTc interval in patient III.4 supports the conclusion that the increase in I_Ca,L_ plays a pathophysiological relevant role in this patient. In healthy controls, diltiazem therapy revealed no QTc‐shortening effect previously (Ritterman *et al*, 1982), supporting our experimental results that control hiPSC‐CMs showed less effect upon diltiazem. A previous publication revealed a causative *ACTN2* mutation for HCM with a pro‐arrhythmic phenotype (Bagnall *et al*, 2014). Although patients III.4 and II.5 have not suffered from excessive ventricular extrasystole or ventricular tachycardia yet, normalized QTc interval by I_Ca,L_ inhibition might reduce the risk of sudden cardiac death by preventing cellular Ca^2+^‐overload and triggered arrhythmia as shown in a mouse model of HCM (Westermann *et al*, 2006). In a large HCM cohort, the mean QTc was only mildly prolonged (440 ms versus 425 ms in controls) (Johnson *et al*, 2011), but in a subgroup of genetically identified HCM patients with mild phenotype and symptoms, 9% showed a QTc > 480 ms, suggesting that impaired repolarization may be not an uncommon feature of the disease. Disease modeling of several different mutations in hiPSC‐CMs showed mostly abnormal Ca^2+^ handling but shortened, unchanged, or prolonged APD, paralleling the clinical heterogeneity (reviewed in Eschenhagen & Carrier, 2019). Examination of the patient's further clinical course will show whether normalization of the QTc interval as a surrogate of clinical improvement will be meaningful and if diltiazem treatment will positively affect other HCM parameters in this patient.

Taken together, this “case study” revealed a novel, rare HCM mutation associated with a contractile and electrophysiological phenotype in hiPSC‐CMs/‐EHTs that guided clinical therapy in two HCM patients. We believe that this study may pave the way for more personalized approaches for less evident HCM gene or variants of uncertain significance. While pharmacological intervention with diltiazem acutely ameliorated the prolonged QTc, longer‐term follow‐up is necessary to see whether diltiazem also affects progression of structural aspects of the disease. The interesting fact that the prolonged QTc did not find attention during clinical routine but was revealed after *in vitro* studies supports the view for more personalized medicine. Given the relevant frequency of QTc prolongation in HCM and the possibility that other HCM mutations as well as myocardial disarray in a more general sense may affect LTCC activity (Viola & Hool, 2017), future work should be directed toward answering whether the beneficial effects of diltiazem treatment observed in our study and in a previously published pilot study (Ho *et al*, 2015) may in fact relate to a general mechanism of HCM pathology or might be specific due to altered interaction between α‐actinin 2 and the LTCC, as indicated by our BRET analysis and recently identified (Tseng *et al*, 2017).

## Materials and Methods

### Human material

The HCM patient carrying the ACTN2 (c.740C>T; dbSNP ID: rs755492182) mutation was recruited in the outpatient clinic at the University Heart Center Hamburg and provided written informed consent for genetic analysis and the use of fibroblasts. Cardiac tissue of the patient was obtained during septal myectomy. Control donor tissues were from non‐failing human heart tissues not suitable for transplantation or from donors that did not die from cardiac disease but of another cause. All materials were taken with informed consent of the patients and donors. The study was reviewed and approved by the Ethical Committee of the Ärztekammer Hamburg (PV3501), and the index patient gave written informed consent. This study is in accordance with the Code of Ethics of the World Medical Association (Declaration of Helsinki).

### Identification of HCM mutation

Genomic DNA was obtained from peripheral blood by standard procedures, and mutations in 19 known or suspected HCM genes were detected. Gene target enrichment was undertaken using HaloPlex Target Enrichment System (Agilent) and sequencing carried out on an Illumina MiSeq Desktop Sequencer. Sequence data were analyzed using a custom‐designed bioinformatic pipeline. The presence of the mutation was confirmed by Sanger sequencing in DNA derived from blood leukocytes. Segregation analysis of the mutation was also performed by Sanger sequencing according to standard procedures (details are available upon request). For *in silico* pathogenicity prediction, the programs CADD (Combined Annotation Dependent Depletion) (Kircher *et al*, 2014), REVEL (Rare exome variant ensemble learner) (Ioannidis *et al*, 2016), and M‐CAP (Mendelian Clinically Applicable Pathogenicity) (Jagadeesh *et al*, 2016) were used. Pathogenicity thresholds were ≥ 20 (CADD), ≥ 0.3 (REVEL), and ≥ 0.025 (M‐CAP).

### Electrocardiography

Electrocardiographic analysis was performed by using a 12‐lead ECG using an automated analyses program (Schiller Cardiovit AT‐10 plus Felsberg, Germany). All QT and RR intervals were re‐analyzed manually and averaged from by three reviewers independently and blinded for genotype and drug intervention. Mean QT intervals (Table 1) deviate slightly from representative ECG measurements from one reviewer (Fig 4I–K; Appendix Fig S5). QT intervals were measured in lead II or V_5_ when possible and under consideration of all leads. QTc values were calculated using the Bazett (QTcB = QT/(RR^(1/2)^) and Fredericia (QTcF = QT/(RR^(1/3)^) correction (Rautaharju *et al*, 2009; Vandenberk *et al*, 2016). For the index patient, we measured QT intervals in ECGs recorded before the surgical myectomy because of a post‐operative total left bundle branch block (Appendix Fig S5A) which does not allow a valid QT interpretation.

### Measurement of monophasic action potentials

Written informed consent was obtained before the study from the index patient. Bisoprolol had been discontinued for 48 h before the procedure. MAPs were measured after the ablation procedure in stable sinus rhythm. MAPs were recorded at different positions on right ventricular septum by a 4‐pole catheter (EasyMAP, MedFact, Lörrach, Germany) during constant right atrial pacing of 2 Hz as described before (Shimizu *et al*, 1991). Signals of MAPs were recorded at 1 kHz and filtered at a frequency of 0.05–500 Hz (BARD LabSystem, Bard Electrophysiology, Murray Hill, MA).

### Next‐generation sequencing for LQT syndrome

After library preparation by the TruSight™ Rapid Capture Kit and targeted enrichment by the TruSight™ Cardio Kit (Illumina, San Diego, CA), cluster generation and sequencing was performed on a MiSeq flow cell with the MiSeq Reagent Kit v.2 on a MiSeq platform (Illumina) according to the manufacturer's instructions. Data analysis was done by Sequence Pilot module SeqNext (JSI Medical Systems, Kippenheim, Germany) with the reference sequences NM_000719.6 (*CACNA1C*), NM_006888.4 (*CALM1*), NM_033337.2 (*CAV3*), NM_000219.5 (*KCNE1*), NM_172201.1 (*KCNE2*), NM_000238.3 (*KCNH2*), NM_000891.2 (*KCNJ2*), NM_000890.3 (*KCNJ5*), NM_000218.2 (*KCNQ1*), NM_174934.3 (*SCN4B*), NM_198056.2 (*SCN5A*), and NM_003098.2 (*SNTA1*). According to the manufacturer's instructions, Multiplex ligation‐dependent probe amplification (MLPA^®^) was performed with the kit P114‐B2 (MRC Holland) for the genes *KCNE1*,* KCNE2*,* KCNH2*, and *KCNQ1*. Data were analyzed by the software Sequence Pilot module MLPA (JSI Medical Systems) after capillary electrophoresis on an automated capillary sequencer (ABI 3500; Applied Biosystems).

### Generation and culture of hiPSC‐derived cardiomyocytes in 2D and in EHT format

A skin biopsy was taken, washed in PBS, minced, and placed in a 6‐well plate in fibroblast medium [DMEM with 10% FBS (PAA), 2 mM l‐glutamine and 0.5% penicillin and streptomycin (all Life Technologies)]. Dermal fibroblasts growing out of the explants were collected for passaging or cryopreservation and used for subsequent reprogramming at passage 5. The reprogramming was performed according to previously published protocols with retroviruses encoding the human transcription factors OCT3/4, SOX2, KLF4, and L‐MYC (Takahashi *et al*, 2007a,b; Ohnuki *et al*, 2009). Fibroblast from the HCM patient with the *ACTN2* (c.740C>T) mutation was reprogrammed using the CytoTune‐iPS Sendai Reprogramming Kit (Product No. A1377801, Life Technologies). CM differentiation from hiPSCs was performed following a three‐step protocol with generation of embryoid bodies (EBs) in spinner flasks and FACS analysis, using a cardiac troponin T FITC‐labeled antibody (1:10, Miltenyi Biotec) for CMs quantification (Breckwoldt *et al*, 2017; Appendix Fig S6). Dissociation was done with collagenase 2 (200 units/ml, Worthington, LS004176), and beating hiPSC‐CMs were plated on Geltrex‐coated (1:100, Gibco) 96‐well or 12‐well plates at a density of 2,500–5,000 cells/well or 440,000 cells/well, respectively. HiPSC‐CMs were maintained in culture for 30 days at 37°C in 7% CO_2_ and atmospheric O_2_ (21%) prior to further analysis. Furthermore, single‐cell suspensions of hiPSC‐CM were subjected to the 3D format of EHTs, which were generated in a 24‐well format (800,000 hiPSC‐CM/EHT in a fibrin matrix (total volume 100 μl) consisting of 10 μl/100 μl Matrigel (BD Biosciences, 256235), 5 mg/ml bovine fibrinogen [200 mg/ml in NaCl 0.9% (Sigma, F4753) plus 0.5 μg/mg aprotinin (Sigma, A1153), 2× DMEM, 10 μmol/l Y‐27632, and 3 U/ml thrombin (Biopur, BP11101104), as described previously (Mannhardt *et al*, 2016; Breckwoldt *et al*, 2017)]. EHTs were maintained in culture at 37°C in 7% CO_2_ and 40% O_2_, subjected to further analysis from day 26 on. Medium exchange for 2D and EHT cultures was performed every Monday, Wednesday, and Friday using DMEM [10% heat‐inactivated FCS (Gibco), 0.1% insulin (Sigma‐Aldrich), 0.5% penicillin/streptomycin (Gibco), 0.1% Aprotinin (Sigma)]. Silicone racks (C001), Teflon spacers (C002), and EHT analysis equipment (A001) were purchased from EHT Technologies GmbH (Hamburg, Germany).

### Generation of the isogenic control cell line HCMrep

For generation of an isogenic control cell line, patient‐specific hiPSCs carrying the *ACTN2* (c.740C>T) mutation were used, hereafter named HCM cell line. CRISPR/Cas9 technology was used to repair the patient‐specific mutation according to Ran *et al* (2013). In short, Cas9 nickase approach was used for induction of a double‐strand break (DSB) in the desired locus. Single‐guide RNAs (sgRNAs) were designed using a publically available tool (crispr.mit.edu) and cloned into the pSpCas9n(BB)‐2A‐GFP plasmid (Addgene). Homology‐directed repair (HDR) was supported by supplying an exogenous repair template encoding the WT sequence of *ACTN2* from position g.54,148‐54,270 (NG_009081.1; Accession number NCBI). Repair templates were produced by IDT^®^ as single‐stranded oligonucleotide donors (ssODNs). Silent mutations were introduced at genomic positions 54,200 and 54,245 within the protospacer adjacent motif (PAM) sequence for prevention of repeated cutting events and verification of successful genome editing. HCM hiPSCs were transfected with the Amaxa Nucleofector™ (Lonza) using the P3 Primary Cell 4D‐Nucleofector^®^ X Kit and the pulse code CA 137. Each transfection approach consisted of 800,000 HCM hiPSCs, 40 μM of ssODN, 1,000 ng of each plasmid pSpCas9n(BB)‐2A‐GFP (Addgene) containing sgRNA A and B. One hour before transfection, HCM hiPSCs were treated with 10 μM ROCK inhibitor Y‐27632 (Y; Biorbyt). For transfection, HCM hiPSCs were washed twice with PBS and singularized with accutase (Gibco^®^; 5 min, 37°C at 5% CO_2_). Enzymatic dissociation was stopped by adding the same volume of conditioned medium (COM) containing bFGF (30 ng/ml; Peprotech) and 10 μM Y. After counting, 800,000 cells were spun down for 3 min at 150 g and subsequently resuspended in the transfection mix and transferred into electroporation cuvettes (Lonza). After transfection, electroporation cuvettes were incubated for 5 min at 37°C in 5% CO_2_. 500 μl pre‐warmed COM, supplemented with FGF (30 ng/ml) and 10 μM Y, was added to the transfection mix that subsequently was plated onto Matrigel‐coated (1:60; Corning^®^) 12‐well plates. Transfected HCM hiPSCs were maintained for 48 h and prepared for fluorescent activated cell sorting (FACS) by accutase dissociation as described above. FACS was performed using the FACSAria™ III (BD; FACS Core facility UKE Hamburg) collecting GFP‐positive cells. GFP‐positive cells were centrifuged for 3 min at 150 *g*, and 2,500 cells were plated into one well of a Matrigel‐coated 6‐well plate. Cells were maintained by daily medium change until colonies were visible. Picking was carried out using a 200‐μl pipette, whereby each clone was transferred into one well of a Matrigel‐coated 48‐well plate. Clones were maintained until sufficient cell material was generated for cryopreservation and genomic analysis by PCR. If genomic analysis revealed successfully repaired HCM hiPSC clones, these clones were subcloned by plating 1,000 cells into one well of a Matrigel‐coated 6‐well plate. Subcloned repaired HCM hiPSC clones were again picked and cultured, until characterization could be repeated as described above. Furthermore, these clones were analyzed for off‐targets by PCR (Appendix Table S2). Subcloning was introduced to minimize chances of mixed clonal populations. Additionally, PCR fragments of cells containing the modified *ACTN2* locus were subcloned using the CloneJET PCR Cloning Kit (Thermo Fisher Scientific) for discrimination of allele‐specific genotypes. Finally, all used cell lines were genotyped by PCR for the *ACTN2* locus and karyotyped by G‐banding as reported previously (Breckwoldt *et al*, 2017). An overview of this procedure is depicted in Appendix Fig S1A.

### Genotyping and off‐target analysis using polymerase chain reaction

To amplify specific DNA fragments, PCR was conducted according to Mullis (Mullis & Faloona, 1987). The applied PCR program was adapted to the used polymerase and annealing temperatures of the primers (listed in the data sheet supplied with the primers). The elongation time was adjusted to the length of the expected DNA fragment, and the synthesis rate was estimated to be 1 kb/min, unless noted otherwise by the manufacturer. Genomic DNA template was used in an amount of 20–40 ng in a final volume of 20 μl and 50–100 ng in a final volume of 50 μl. All used primers are listed in Appendix Table S3. For genotyping of putative CRISPR/Cas9‐modified HCM hiPSCs clones, touchdown PCR (60°C–55°C) was performed using PrimeStar^®^ HS DNA Polymerase in a 50‐μl PCR approach, for 35 cycles according to the instructions of the manufacturer's protocol. For off‐target analysis of successfully repaired HCM hiPSCs clones, touchdown PCR (60°C–55°C) was performed using AmpliTaq^®^ Gold DNA polymerase in a total volume of 20 μl for 35 cycles according to the instructions of the manufacturer's protocol.

### Analysis of allele‐specific mRNA transcripts

Quantification of missense and WT mRNA was done with total RNA of EHTs and of myectomy tissue received from the index patient. RNA (200 ng) was reverse‐transcribed to cDNA according to the instructions of the manufacturer's protocol (SuperScript™ III First‐Strand Synthesis System, Invitrogen). cDNA was amplified with PrimeStar^®^ HS DNA Polymerase in a 50‐μl PCR approach, for 35 cycles according to the instructions of the manufacturer's protocol (Primers, Appendix Table S3). Generated PCR fragments were purified with the QIAquick PCR Purification Kit (Qiagen) and cloned with the CloneJET PCR Cloning Kit (Thermo Fisher Scientific) for discrimination of allele‐specific mRNA transcripts. Single colonies of transformed One Shot^®^ TOP10 *E. coli* were transferred to overnight cultures [5 ml, lysogeny broth (BD), ampicillin (100 μg/ml; Serva)]. Plasmid extraction (NucleoSpin^®^ Plasmid, Macheray‐Nagel) was performed the next day and send for sequencing (Eurofins Genomics).

### RNA isolation and gene expression analysis

Total RNA was extracted from 2D‐cultured hiPSC‐CMs and ‐EHTs using TRIzol^®^ Reagent (Life Technologies) following the manufacturer's protocol. RNA extraction from human heart tissue was performed with the SV total RNA isolation Kit (Promega). For RNA sequencing analysis, RNA integrity was analyzed with the RNA 6000 Nano Chip on an Agilent 2100 Bioanalyzer (Agilent Technologies). From total RNA, mRNA was extracted using the NEBNext Poly(A) mRNA Magnetic Isolation module (New England Biolabs) and RNA‐Seq libraries were generated using the NEXTFLEX Rapid Directional qRNA‐Seq Kit (Bioo Scientific) as per the manufacturer's recommendations. Concentrations of all samples were measured with a Qubit 2.0 Fluorometer (Thermo Fisher Scientific), and fragment lengths distribution of the final libraries was analyzed with the DNA High Sensitivity Chip on an Agilent 2100 Bioanalyzer (Agilent Technologies). All samples were normalized to 2 nM and pooled equimolar. The library pool was sequenced on the NextSeq 500 (Illumina) with 1 × 75 bp, with 16.6–23.8 mio. reads per sample. For each sample, the sufficient quality of the raw reads was confirmed by FastQC v0.11.8 (http://www.bioinformatics.babraham.ac.uk/projects/fastqc). Afterward, the reads were aligned to the human reference genome GRCh38 with STAR v2.7.0f (Dobin *et al*, 2013) and simultaneously counted per gene by employing the *quantmode GeneCounts* option. Counts are based on the Ensembl annotation release 96. Normalization factors for each sample were estimated with *DESeq2* v1.24.0 (Love *et al*, 2014) and used to generate normalized coverage tracks with *bamCoverage* from *deepTools* v3.2.1 (Ramirez *et al*, 2016). Mutations and expressions were visualized with *Gviz* v1.28.0 (Hahne & Ivanek, 2016). For expression analysis with the nanoString nCounter^®^ Elements technology, a total amount of 50 ng RNA was hybridized with a customized nanoString Gene Expression CodeSet (Appendix Table S4) and analyzed using the nCounter^®^ Sprint Profiler. mRNA levels were determined with the nSolver™ Data Analysis Software including background subtraction using negative controls and normalization to six or four (2D‐cultured hiPSC‐CMs and EHTs, indicated in Appendix Fig S2D) housekeeping genes (*ABCF1, CLTC, GAPDH, ACTB, PKG1, TUBB*; Appendix Table S4) and expressed as fold change over Ctrl for the respective groups.

### Western Blot analysis

Western blot analysis was performed on total crude protein lysates from human tissue, 2D‐cultured hiPSC‐CMs, and EHTs. Lysates of single samples or pooled samples were separated on 10% acrylamide/bisacrylamide (29:1) gels and transferred by wet electroblotting to nitrocellulose membranes. Membranes were stained with primary antibodies directed against α‐actinin 2 (1:1,000, Sigma) and cardiac troponin T (1:5,000, Abcam). Peroxidase‐conjugated secondary antibodies against mouse (1:5,000, Sigma) or against rabbit (1:6,000, Sigma) were used. Signals were revealed with the Clarity Western ECL Substrate (Bio‐Rad) and acquired with the ChemiDoc Touch Imaging System (Bio‐Rad). Signals were quantified with the Image Lab software (Bio‐Rad).

### Immunofluorescence staining of hiPSC‐derived cardiomyocytes

HiPSC‐CMs were cultured for 30 days in 96‐well plates (μclear^®^, Greiner) and subsequently prepared for immunofluorescent analysis, as described previously (Prondzynski *et al*, 2017). Staining for α‐actinin 2 was performed by a primary antibody (1:800, Sigma) and a secondary anti‐mouse Alexa Fluor^®^ 488 antibody (1:800, Life Technologies). The nucleus was stained with Hoechst 33342 (1:2,500, Thermo Fisher Scientific), and images were obtained by confocal microscopy using the Zeiss LSM 800 confocal microscope.

### Morphological analysis of 2D‐cultured hiPSC‐derived cardiomyocytes

Quantification of myofibrillar disarray and cell area was done by using Fiji software (ImageJ), whereby cell area was assessed as described previously (Prondzynski *et al*, 2017). Myofibrillar disarray was measured by drawing randomly three regions of interests (ROI) in single hiPSC‐CMs cultured in 96‐well plates. Two lines were drawn with a length of approximately 10 μm for each ROI, spanning ~6 sarcomere intervals, perpendicular to the sarcomere alignment in a distance of 5–10 μm. Myofibrillar disarray was quantified by dividing the parallel intersections of the sarcomere with the two lines and quantifying the standard deviation of each crossed intersection, resulting in the index of myofibrillar disarray. Analysis of myofibrillar disarray was performed in a blinded fashion.

### Analysis of contractile force in EHTs

Contractile force was analyzed as previously described (Mannhardt *et al*, 2016). In short, > 26‐day‐old EHTs were incubated in modified Tyrode's solution at least 2 h before starting an experiment (in mM: NaCl 120, KCl 5.4, MgCl_2_ 1, CaCl_2_ 1.8, NaH_2_PO_4_ 0.4, NaHCO_3_ 22.6, glucose 5, Na_2_EDTA 0.05, and HEPES 25) and incubated at 37°C in 7% CO_2_ and 40% O_2_. The 24‐well plate carrying the EHTs was placed inside a transparent chamber to maintain homeostatic temperature (37°C) and CO_2_ (5%). Automated video‐optical recordings of silicon post deflection were enabled by a video camera placed above the chamber. Tracking of EHT movement and determination of contractile force was analyzed based on the known mechanical properties of the silicon using customized software (CTMV, Pforzheim, Germany). EHTs were electrically paced for experimental analysis (1 V, 2 Hz, impulse duration 4 ms). The contraction peaks were analyzed for force amplitude, contraction time (T1_80%_), and relaxation time (T2_80%_) at 80% of peak height.

### Myofilament Ca^2+^ sensitivity measurements of human tissue and EHTs

Cardiac muscle strips of 2.69 ± 0.82 mm length, 0.32 ± 0.08 mm width, and 0.20 ± 0.09 mm^2^ cross‐sectional area (CSA), calculated by 2πr^2^ assuming a circular shape, were isolated from human cardiac tissues and EHTs. For contractile measurements, strips were permeabilized in a pCa9 EGTA‐buffer containing 1% Triton X‐100 at 4°C for 18 as described previously (Kooij *et al*, 2010; Stoehr *et al*, 2014; Friedrich *et al*, 2016). The next day strips were either used directly for measurements or stored at −20°C in a 50% glycerol/relaxing solution with protease inhibitors (EDTA‐free, complete tablets, mini, Roche). Measurement of contractile function was performed using a fiber test system (1400A; Aurora Scientific) as reported previously (Friedrich *et al*, 2016; Stucker *et al*, 2017). A Hill equation was fitted to the data points (Hill *et al*, 1980) to estimate pCa_50_ as the free Ca^2+^ concentration yielding 50% of the maximal force and nH representing the Hill coefficient. The pCa_50_ represents the parameter of myofilament Ca^2+^ sensitivity.

### Action potential measurements of EHTs

APs were recorded as described previously (Wettwer *et al*, 2013; Lemoine *et al*, 2017) with standard sharp microelectrodes in intact EHTs, field‐stimulated at 1 Hz (50% above the stimulation threshold). Tissues were continuously superfused with Tyrode's solution containing (in mM): NaCl 127, KCl 5.4, MgCl_2_ 1.05, CaCl_2_ 1.8, glucose 10, NaHCO_3_ 22, NaHPO_4_ 0.42, equilibrated with O_2_‐CO_2_ [95:5] at 36°C, pH 7.4. Sharp microelectrodes were made from filamented borosilicate glass capillaries (external diameter 1.5 mm, internal diameter 0.87 mm; HILG1103227; Hilgenberg, Malsfeld, Germany) by a DPZ‐Universal puller (Zeitz Instruments, Munich, Germany). The pipettes had a resistance of 20–50 MΩ when filled with 3 M KCl. Pipettes were mounted on a hydraulic micromanipulator (Narishige MO‐203) for gentle impalement in the tissue. Signals were amplified by BA‐1s npi amplifier (npi electronic, Tamm, Germany). EHTs were detached from the silicon posts and pinned down without any stretching. To measure rate adaptation, we allowed preparations to equilibrate for at least 300 beats. To measure diltiazem effect, we allowed preparations to equilibrate for at least 10 min after drug exposure. APs were recorded and analyzed using the Lab Chart software (ADInstruments, Spechbach, Germany).

### Whole‐cell recording of I_Ca,L_

The L‐type calcium current (I_Ca,L_) was measured in the whole‐cell configuration of the patch clamp technique by an Axopatch 200B amplifier (Axon Instruments, Foster City, CA, USA) at 37°C. The experimental procedure followed a previous publication (Uzun *et al*, 2016). The ISO2 software was used for data acquisition and analysis (MFK, Niedernhausen, Germany). Heat‐polished pipettes were used. Cell capacitance (C_m_) was calculated from steady‐state current during depolarizing ramp pulses (1 V/1 s) from −40 to −35 mV. Ca^2+^ currents were elicited by applying test pulses from −80 to +10 mV (0.5 Hz). The cells were investigated in a small perfusion chamber placed on the stage of an inverse microscope. In order to avoid contaminating currents, K^+^ currents were blocked by replacing K^+^ with Cs^+^ and tetraethylammonium chloride in the bath solution. The experiments were performed with the following Na^+^‐free bath solution (in mM): tetraethylammonium chloride 120, CsCl 10, HEPES 10, CaCl_2_ 2, MgCl_2_ 1, and glucose 20 (pH 7.4, adjusted with CsOH) at 37°C. The pipette solution (pH 7.2, adjusted with CsOH) included (in mM): cesium methanesulfonate 90, CsCl 20, HEPES 10, Mg‐ATP 4, Tris‐GTP 0.4, EGTA 10, and CaCl_2_ 3 (Christ *et al*, 2001). Current amplitude was determined as the difference between peak inward current and current at the end of the depolarizing step. The selective I_CaL_‐blocker nifedipine (10 μM) was used to identify I_Ca,L_.

### Bioluminescence resonance energy transfer (BRET)

BRET assays were performed in HL‐1 cardiac cells as previously described (Rusconi *et al*, 2016). Briefly, Ca_v_α1.2 and *ACTN2* (WT and mutant) cDNAs were cloned into the pNLF1‐N and the HaloTag‐pFN21A vector (Promega), respectively, and then transfected in HL‐1 cells in a Ca_v_α1.2‐NanoLuc:*ACTN2*‐Halo (WT or mutant) 1:100 ratio. Ca_v_β2 was added as indicated. Forty‐eight hours after transfections, cells were treated with 100 nmol/l NanoBRET 618 ligand, and signals were detected 5 h after treatment, with a Synergy 4 instrument (BioTek).

### Mathematical modeling and computer simulations of cardiomyocyte function

To simulate the effect of altered I_Ca,L_ density on the AP and force development, we updated our previously used hiPSC‐like computational CM model (Lemoine *et al*, 2018) to include the description of the contractile element, as implemented by Ji *et al* (2015) based on the original formulation from Negroni and Lascano (1996). The HCM‐related change in the I_Ca,L_ amplitude, which was observed in the patch clamp experiments, was accounted for by increasing the maximum conductance by 50% in the computational CM model. Simulation of electrophysiological and contractile function of the CM and post‐processing was done with the commercial MATLAB software (release 2017b, https://www.mathworks.com/products/matlab.html), using the built‐in function (ode15s) to solve the differential equations.

### Antibodies

All antibodies were commercially available, validated, and suitable for human species as specified by the manufacturer: Monoclonal anti‐α‐actinin used in a 1:800 dilution for immunofluorescence staining (Sigma, produced in mouse, Cat.: A7811‐.2ML, Lot:027M4813V); goat anti‐mouse IgG (H+L) highly cross‐adsorbed secondary antibody; Alexa Fluor 488 used in a 1:800 dilution for immunofluorescence staining (Invitrogen, produced in goat, Cat.: A‐11029, Lot:1829920); anti‐ACTN2 used in a 1:1,000 dilution for Western blot analysis (Sigma, produced in rabbit, Cat.: SAB2108642‐100UL, Lot: QC12269); anti‐cardiac troponin T antibody used in a 1:5,000 dilution for Western blot analysis (Abcam, produced in mouse, Cat.: ab8295, Lot: GR3232545‐1); anti‐mouse IgG (whole molecule)–peroxidase antibody used in a 1:5,000 dilution for Western blot analysis (Sigma, produced in rabbit, Cat.: A9044‐2ML, Lot: 055M4818V); anti‐rabbit IgG (whole molecule)–peroxidase antibody used in a 1:6,000 dilution for Western blot analysis (Sigma, produced in goat, Cat.: A0545‐1ML, Lot: 065M4769V); anti‐cardiac troponin T‐FITC used in a 1:10 dilution for FACS analysis (Miltenyi Biotec, Cat.: 130‐119‐575, Lot: 5181219151); and REA‐control (I)‐FITC used in a 1:10 dilution for FACS analysis as isotype control (Miltenyi Biotec, Cat.: 130‐104‐611, Lot: 5180809130).

### Statistics

GraphPad Prism 5 and 8 (GraphPad Software, San Diego, CA, USA) was used for data analyses. Curves were fitted to data points from individual experiments, and exact number of replicates is indicated in figure legends. Data were compared using unpaired/paired Student's *t*‐test, or using two‐way ANOVA followed by Sidak's multiple comparisons test when appropriate, and for more than two groups one‐way ANOVA followed by Bonferroni's post‐test, if not indicated differently. All analyses were two‐tailed, and a *P *<* *0.05 was considered as statistically significant, whereby exact *P*‐values are indicated in graphs. Values are expressed as mean ± SEM. For myofilament Ca^2+^ sensitivity measurements, concentration–response curves were fitted to the data points and force–pCa relationship comparison was done by using extra sum‐of‐squares *F*‐test.

## Data and materials availability

Data, analytic methods, and study materials will be made available on request to other researchers for purposes of reproducing the results or replicating the procedures within the limits required for patient confidentiality.

## Author contributions

Conception and design of the work: MPr, MDL, TE, and LC; Methodology: MPr and MDL; Acquisition, analysis, and/or interpretation of the data: MPr, MDL, ATLZ, AH, VdM, JTK, NK, JB, TK, EK, SS, MS, FWF, JM, SDL, CR, AEV, AHa, GM, DC, CM, TC, MPa, TE, and LC; Patient recruitment: JM, MDL, and MPa; Original draft: MPr and MDL; Review and editing: MPr, MDL, DC, TC, TE, and LC; Funding acquisition, MPr, MDL, AHa, TC, TE, and LC. All authors participated in the critical review of the manuscript and approved the submitted version.

## Conflict of interest

A.Ha. and T.E. are cofounders of EHT Technologies GmbH, Hamburg. All the other authors declare no conflict of interest.

**The paper explained**

**Problem**

Hypertrophic cardiomyopathy (HCM) is the most prevalent inherited heart disease accompanied by structural and contractile alterations. The genetic diversity is paralleled by clinical heterogeneity, highlighting the need for individualized treatment approaches in HCM. The goal of this study was to elucidate the functional role of a novel mutation in *ACTN2*, encoding α‐actinin 2, in human‐induced pluripotent stem cell (hiPSC)‐derived cardiomyocytes and translate *in vitro* findings to the affected family.

**Results**

We identified a rare *ACTN2* c.740C>T transition (p.T247M) in an HCM patient, her son, and sister. To identify underlying cellular pathologies, patient‐derived hiPSC and CRISPR/Cas9‐derived isogenic control were generated. Compared to control, HCM hiPSC‐derived cardiomyocytes/engineered heart tissues recapitulated several hallmarks of HCM, such as myofibrillar disarray, hypercontractility, prolonged relaxation, and higher myofilament Ca^2+^ sensitivity. Additionally, L‐type calcium channel (LTCC) current density was higher and action potential duration (APD) was longer in HCM than in control. Mutant α‐actinin 2 exhibited reduced interaction with the LTCC complex. The LTCC blocker diltiazem reduced force amplitude, relaxation, and APD to a greater extent in HCM than in control hiPSC‐engineered heart tissue. We translated our findings to patient care and showed that diltiazem ameliorated the prolonged QTc interval in the HCM‐affected son and sister of the index patient.

**Impact**

This study revealed a novel HCM mutation associated with a contractile and electrophysiological phenotype in hiPSC‐derived cardiomyocytes and engineered heart tissues and a genotype‐specific pathomechanism that guided clinical therapy in a family with HCM. Long‐term follow‐up is necessary to see whether diltiazem also prevents development of arrhythmia and progression of structural aspects of the disease. This study may serve as proof‐of‐principle for the use of hiPSC‐cardiomyocytes and CRISPR/Cas9 for personalized treatment of cardiomyopathies.

## For more information

(i) https://omim.org/entry/102573

(ii) https://www.mayoclinic.org/diseases-conditions/hypertrophic-cardiomyopathy/symptoms-causes/syc-20350198

(iii) https://www.heart.org/en/health-topics/cardiomyopathy/what-is-cardiomyopathy-in-adults/hypertrophic-cardiomyopathy

(iv)http://www.eht-technologies.com/

(v)https://dzhk.de/en/

(vi)https://www.ndr.de/ratgeber/gesundheit/UKE-Hamburg-Fortschritt-in-der-Herzforschung,herz588.html

## Supporting information



Appendix

Movie EV1

Movie EV2

Movie EV3

Movie EV4

Source Data for Appendix

Review Process File

Source Data for Figure 1

## References

[emmm201911115-bib-0001] Adabag AS , Casey SA , Kuskowski MA , Zenovich AG , Maron BJ (2005) Spectrum and prognostic significance of arrhythmias on ambulatory Holter electrocardiogram in hypertrophic cardiomyopathy. J Am Coll Cardiol 45: 697–704 15734613 10.1016/j.jacc.2004.11.043

[emmm201911115-bib-0002] Bagnall RD , Molloy LK , Kalman JM , Semsarian C (2014) Exome sequencing identifies a mutation in the ACTN2 gene in a family with idiopathic ventricular fibrillation, left ventricular noncompaction, and sudden death. BMC Med Genet 15: 99 25224718 10.1186/s12881-014-0099-0 PMC4355500

[emmm201911115-bib-0003] Breckwoldt K , Letuffe‐Breniere D , Mannhardt I , Schulze T , Ulmer B , Werner T , Benzin A , Klampe B , Reinsch MC , Laufer S *et al* (2017) Differentiation of cardiomyocytes and generation of human engineered heart tissue. Nat Protoc 12: 1177–1197 28492526 10.1038/nprot.2017.033

[emmm201911115-bib-0004] Chiu C , Bagnall RD , Ingles J , Yeates L , Kennerson M , Donald JA , Jormakka M , Lind JM , Semsarian C (2010) Mutations in alpha‐actinin‐2 cause hypertrophic cardiomyopathy: a genome‐wide analysis. J Am Coll Cardiol 55: 1127–1135 20022194 10.1016/j.jacc.2009.11.016

[emmm201911115-bib-0005] Chopra A , Kutys ML , Zhang K , Polacheck WJ , Sheng CC , Luu RJ , Eyckmans J , Hinson JT , Seidman JG , Seidman CE *et al* (2018) Force generation via beta‐cardiac myosin, titin, and alpha‐actinin drives cardiac sarcomere assembly from cell‐matrix adhesions. Dev Cell 44: 87–96 e85 29316444 10.1016/j.devcel.2017.12.012 PMC6421364

[emmm201911115-bib-0006] Christ T , Wettwer E , Dobrev D , Adolph E , Knaut M , Wallukat G , Ravens U (2001) Autoantibodies against the beta1 adrenoceptor from patients with dilated cardiomyopathy prolong action potential duration and enhance contractility in isolated cardiomyocytes. J Mol Cell Cardiol 33: 1515–1525 11448139 10.1006/jmcc.2001.1414

[emmm201911115-bib-0007] Dambrot C , Braam SR , Tertoolen LG , Birket M , Atsma DE , Mummery CL (2014) Serum supplemented culture medium masks hypertrophic phenotypes in human pluripotent stem cell derived cardiomyocytes. J Cell Mol Med 18: 1509–1518 24981391 10.1111/jcmm.12356 PMC4190898

[emmm201911115-bib-0008] Dobin A , Davis CA , Schlesinger F , Drenkow J , Zaleski C , Jha S , Batut P , Chaisson M , Gingeras TR (2013) STAR: ultrafast universal RNA‐seq aligner. Bioinformatics 29: 15–21 23104886 10.1093/bioinformatics/bts635 PMC3530905

[emmm201911115-bib-0009] Eden M , Meder B , Volkers M , Poomvanicha M , Domes K , Branchereau M , Marck P , Will R , Bernt A , Rangrez A *et al* (2016) Myoscape controls cardiac calcium cycling and contractility via regulation of L‐type calcium channel surface expression. Nat Commun 7: 11317 27122098 10.1038/ncomms11317 PMC5438100

[emmm201911115-bib-0010] Eschenhagen T (2018) Treatment of ischemic heart disease In Goodman & Gilman's the phamarcological basis of therapeutics, Brunton LL, Hilal‐Dandan R, Knollmann BC (eds), pp 489–506. New York, NY: McGraw‐Hill Education

[emmm201911115-bib-0011] Eschenhagen T , Carrier L (2019) Cardiomyopathy phenotypes in human‐induced pluripotent stem cell‐derived cardiomyocytes‐a systematic review. Pflugers Arch 471: 755–768 30324321 10.1007/s00424-018-2214-0 PMC6475632

[emmm201911115-bib-0012] Fraysse B , Weinberger F , Bardswell SC , Cuello F , Vignier N , Geertz B , Starbatty J , Kramer E , Coirault C , Eschenhagen T *et al* (2012) Increased myofilament Ca^2+^ sensitivity and diastolic dysfunction as early consequences of Mybpc3 mutation in heterozygous knock‐in mice. J Mol Cell Cardiol 52: 1299–1307 22465693 10.1016/j.yjmcc.2012.03.009 PMC3370652

[emmm201911115-bib-0013] Friedrich FW , Flenner F , Nasib M , Eschenhagen T , Carrier L (2016) Epigallocatechin‐3‐gallate accelerates relaxation and Ca(^2+^) transient decay and desensitizes myofilaments in healthy and Mybpc3‐targeted knock‐in cardiomyopathic mice. Front Physiol 7: 607 27994558 10.3389/fphys.2016.00607 PMC5136558

[emmm201911115-bib-0014] Gautel M , Djinovic‐Carugo K (2016) The sarcomeric cytoskeleton: from molecules to motion. J Exp Biol 219: 135–145 26792323 10.1242/jeb.124941

[emmm201911115-bib-0015] Girolami F , Iascone M , Tomberli B , Bardi S , Benelli M , Marseglia G , Pescucci C , Pezzoli L , Sana ME , Basso C *et al* (2014) Novel alpha‐actinin 2 variant associated with familial hypertrophic cardiomyopathy and juvenile atrial arrhythmias: a massively parallel sequencing study. Circ Cardiovasc Genet 7: 741–750 25173926 10.1161/CIRCGENETICS.113.000486

[emmm201911115-bib-0016] Hahne F , Ivanek R (2016) Visualizing genomic data using gviz and bioconductor. Methods Mol Biol 1418: 335–351 27008022 10.1007/978-1-4939-3578-9_16

[emmm201911115-bib-0017] Hall DD , Dai S , Tseng PY , Malik Z , Nguyen M , Matt L , Schnizler K , Shephard A , Mohapatra DP , Tsuruta F *et al* (2013) Competition between alpha‐actinin and Ca(2)(+)‐calmodulin controls surface retention of the L‐type Ca(2)(+) channel Ca(V)1.2. Neuron 78: 483–497 23664615 10.1016/j.neuron.2013.02.032 PMC4570828

[emmm201911115-bib-0018] Haywood NJ , Wolny M , Rogers B , Trinh CH , Shuping Y , Edwards TA , Peckham M (2016) Hypertrophic cardiomyopathy mutations in the calponin‐homology domain of ACTN2 affect actin binding and cardiomyocyte Z‐disc incorporation. Biochem J 473: 2485–2493 27287556 10.1042/BCJ20160421 PMC4980809

[emmm201911115-bib-0019] Hill TL , Eisenberg E , Greene L (1980) Theoretical model for the cooperative equilibrium binding of myosin subfragment 1 to the actin‐troponin‐tropomyosin complex. Proc Natl Acad Sci USA 77: 3186–3190 10627230 10.1073/pnas.77.6.3186 PMC349579

[emmm201911115-bib-0020] Ho CY (2010) Hypertrophic cardiomyopathy. Heart Fail Clin 6: 141–159 20347784 10.1016/j.hfc.2009.12.001 PMC3031113

[emmm201911115-bib-0021] Ho CY , Lakdawala NK , Cirino AL , Lipshultz SE , Sparks E , Abbasi SA , Kwong RY , Antman EM , Semsarian C , Gonzalez A *et al* (2015) Diltiazem treatment for pre‐clinical hypertrophic cardiomyopathy sarcomere mutation carriers: a pilot randomized trial to modify disease expression. JACC Heart Fail 3: 180–188 25543971 10.1016/j.jchf.2014.08.003 PMC4323670

[emmm201911115-bib-0022] Hong T , Shaw RM (2017) Cardiac T‐tubule microanatomy and function. Physiol Rev 97: 227–252 27881552 10.1152/physrev.00037.2015 PMC6151489

[emmm201911115-bib-0023] Ioannidis NM , Rothstein JH , Pejaver V , Middha S , McDonnell SK , Baheti S , Musolf A , Li Q , Holzinger E , Karyadi D *et al* (2016) REVEL: an ensemble method for predicting the pathogenicity of rare missense variants. Am J Hum Genet 99: 877–885 27666373 10.1016/j.ajhg.2016.08.016 PMC5065685

[emmm201911115-bib-0024] Jagadeesh KA , Wenger AM , Berger MJ , Guturu H , Stenson PD , Cooper DN , Bernstein JA , Bejerano G (2016) M‐CAP eliminates a majority of variants of uncertain significance in clinical exomes at high sensitivity. Nat Genet 48: 1581–1586 27776117 10.1038/ng.3703

[emmm201911115-bib-0025] Ji YC , Gray RA , Fenton FH (2015) Implementation of contraction to electrophysiological ventricular myocyte models, and their quantitative characterization via post‐extrasystolic potentiation. PLoS ONE 10: e0135699 26317204 10.1371/journal.pone.0135699 PMC4552858

[emmm201911115-bib-0026] Johnson JN , Grifoni C , Bos JM , Saber‐Ayad M , Ommen SR , Nistri S , Cecchi F , Olivotto I , Ackerman MJ (2011) Prevalence and clinical correlates of QT prolongation in patients with hypertrophic cardiomyopathy. Eur Heart J 32: 1114–1120 21345853 10.1093/eurheartj/ehr021 PMC3086898

[emmm201911115-bib-0027] Kircher M , Witten DM , Jain P , O'Roak BJ , Cooper GM , Shendure J (2014) A general framework for estimating the relative pathogenicity of human genetic variants. Nat Genet 46: 310–315 24487276 10.1038/ng.2892 PMC3992975

[emmm201911115-bib-0028] Kooij V , Boontje N , Zaremba R , Jaquet K , dos Remedios C , Stienen GJ , van der Velden J (2010) Protein kinase C alpha and epsilon phosphorylation of troponin and myosin binding protein C reduce Ca^2+^ sensitivity in human myocardium. Basic Res Cardiol 105: 289–300 19655190 10.1007/s00395-009-0053-z PMC2807945

[emmm201911115-bib-0029] Lam CK , Tian L , Belbachir N , Wnorowski A , Shrestha R , Ma N , Kitani T , Rhee JW , Wu JC (2019) Identifying the transcriptome signatures of calcium channel blockers in human induced pluripotent stem cell‐derived cardiomyocytes. Circ Res 125: 212–222 31079550 10.1161/CIRCRESAHA.118.314202 PMC6610656

[emmm201911115-bib-0030] Lan F , Lee AS , Liang P , Sanchez‐Freire V , Nguyen PK , Wang L , Han L , Yen M , Wang Y , Sun N *et al* (2013) Abnormal calcium handling properties underlie familial hypertrophic cardiomyopathy pathology in patient‐specific induced pluripotent stem cells. Cell Stem Cell 12: 101–113 23290139 10.1016/j.stem.2012.10.010 PMC3638033

[emmm201911115-bib-0031] Lee KS , Tsien RW (1983) Mechanism of calcium channel blockade by verapamil, D600, diltiazem and nitrendipine in single dialysed heart cells. Nature 302: 790–794 6302512 10.1038/302790a0

[emmm201911115-bib-0032] Lemoine MD , Mannhardt I , Breckwoldt K , Prondzynski M , Flenner F , Ulmer B , Hirt MN , Neuber C , Horvath A , Kloth B *et al* (2017) Human iPSC‐derived cardiomyocytes cultured in 3D engineered heart tissue show physiological upstroke velocity and sodium current density. Sci Rep 7: 5464 28710467 10.1038/s41598-017-05600-w PMC5511281

[emmm201911115-bib-0033] Lemoine MD , Krause T , Koivumaki JT , Prondzynski M , Schulze ML , Girdauskas E , Willems S , Hansen A , Eschenhagen T , Christ T (2018) Human induced pluripotent stem cell‐derived engineered heart tissue as a sensitive test system for QT prolongation and arrhythmic triggers. Circ Arrhythm Electrophysiol 11: e006035 29925535 10.1161/CIRCEP.117.006035

[emmm201911115-bib-0034] Love MI , Huber W , Anders S (2014) Moderated estimation of fold change and dispersion for RNA‐seq data with DESeq2. Genome Biol 15: 550 25516281 10.1186/s13059-014-0550-8 PMC4302049

[emmm201911115-bib-0035] Lu L , Zhang Q , Timofeyev V , Zhang Z , Young JN , Shin HS , Knowlton AA , Chiamvimonvat N (2007) Molecular coupling of a Ca^2+^‐activated K+ channel to L‐type Ca^2+^ channels via alpha‐actinin2. Circ Res 100: 112–120 17110593 10.1161/01.RES.0000253095.44186.72

[emmm201911115-bib-0036] Mannhardt I , Breckwoldt K , Letuffe‐Breniere D , Schaaf S , Schulz H , Neuber C , Benzin A , Werner T , Eder A , Schulze T *et al* (2016) Human engineered heart tissue: analysis of contractile force. Stem Cell Rep 7: 29–42 10.1016/j.stemcr.2016.04.011 PMC4944531 27211213

[emmm201911115-bib-0037] Marian AJ (2009) Contemporary treatment of hypertrophic cardiomyopathy. Tex Heart Inst J 36: 194–204 19568388 PMC2696493

[emmm201911115-bib-0038] Marian AJ , Braunwald E (2017) Hypertrophic cardiomyopathy: genetics, pathogenesis, clinical manifestations, diagnosis, and therapy. Circ Res 121: 749–770 28912181 10.1161/CIRCRESAHA.117.311059 PMC5654557

[emmm201911115-bib-0039] Maron BJ , Gardin JM , Flack JM , Gidding SS , Kurosaki TT , Bild DE (1995) Prevalence of hypertrophic cardiomyopathy in a general population of young adults. Echocardiographic analysis of 4111 subjects in the CARDIA Study. coronary artery risk development in (young) adults. Circulation 92: 785–789 7641357 10.1161/01.cir.92.4.785

[emmm201911115-bib-0040] Maruoka ND , Steele DF , Au BP , Dan P , Zhang X , Moore ED , Fedida D (2000) alpha‐actinin‐2 couples to cardiac Kv1.5 channels, regulating current density and channel localization in HEK cells. FEBS Lett 473: 188–194 10812072 10.1016/s0014-5793(00)01521-0

[emmm201911115-bib-0041] Mosqueira D , Mannhardt I , Bhagwan JR , Lis‐Slimak K , Katili P , Scott E , Hassan M , Prondzynski M , Harmer SC , Tinker A *et al* (2018) CRISPR/Cas9 editing in human pluripotent stem cell‐cardiomyocytes highlights arrhythmias, hypocontractility, and energy depletion as potential therapeutic targets for hypertrophic cardiomyopathy. Eur Heart J 39: 3879–3892 29741611 10.1093/eurheartj/ehy249 PMC6234851

[emmm201911115-bib-0042] Mullis KB , Faloona FA (1987) Specific synthesis of DNA *in vitro* via a polymerase‐catalyzed chain reaction. Methods Enzymol 155: 335–350 3431465 10.1016/0076-6879(87)55023-6

[emmm201911115-bib-0043] Negroni JA , Lascano EC (1996) A cardiac muscle model relating sarcomere dynamics to calcium kinetics. J Mol Cell Cardiol 28: 915–929 8762031 10.1006/jmcc.1996.0086

[emmm201911115-bib-0044] Ohnuki M , Takahashi K , Yamanaka S (2009) Generation and characterization of human induced pluripotent stem cells. Curr Protoc Stem Cell Biol Chapter 4: Unit 4A.2 10.1002/9780470151808.sc04a02s9 19536759

[emmm201911115-bib-0045] Ojala M , Prajapati C , Polonen RP , Rajala K , Pekkanen‐Mattila M , Rasku J , Larsson K , Aalto‐Setala K (2016) Mutation‐specific phenotypes in hiPSC‐derived cardiomyocytes carrying either myosin‐binding protein C Or alpha‐tropomyosin mutation for hypertrophic cardiomyopathy. Stem Cells Int 2016: 1684792 27057166 10.1155/2016/1684792 PMC4707351

[emmm201911115-bib-0046] Prondzynski M , Kramer E , Laufer SD , Shibamiya A , Pless O , Flenner F , Muller OJ , Munch J , Redwood C , Hansen A *et al* (2017) Evaluation of MYBPC3 trans‐splicing and gene replacement as therapeutic options in human iPSC‐derived cardiomyocytes. Mol Ther Nucleic Acids 7: 475–486 28624223 10.1016/j.omtn.2017.05.008 PMC5458066

[emmm201911115-bib-0047] Ramirez F , Ryan DP , Gruning B , Bhardwaj V , Kilpert F , Richter AS , Heyne S , Dundar F , Manke T (2016) deepTools2: a next generation web server for deep‐sequencing data analysis. Nucleic Acids Res 44: W160–W165 27079975 10.1093/nar/gkw257 PMC4987876

[emmm201911115-bib-0048] Ran FA , Hsu PD , Wright J , Agarwala V , Scott DA , Zhang F (2013) Genome engineering using the CRISPR‐Cas9 system. Nat Protoc 8: 2281–2308 24157548 10.1038/nprot.2013.143 PMC3969860

[emmm201911115-bib-0049] Rautaharju PM , Surawicz B , Gettes LS , Bailey JJ , Childers R , Deal BJ , Gorgels A , Hancock EW , Josephson M , Kligfield P *et al* (2009) AHA/ACCF/HRS recommendations for the standardization and interpretation of the electrocardiogram: part IV: the ST segment, T and U waves, and the QT interval: a scientific statement from the American Heart Association Electrocardiography and Arrhythmias Committee, Council on Clinical Cardiology; the American College of Cardiology Foundation; and the Heart Rhythm Society. Endorsed by the International Society for Computerized Electrocardiology. J Am Coll Cardiol 53: 982–991 19281931 10.1016/j.jacc.2008.12.014

[emmm201911115-bib-0050] Ritterman JB , Hossack KF , Bruce RA (1982) Acute and chronic effects of diltiazem on A‐V conduction at rest and during exercise. J Electrocardiol 15: 41–46 7040581 10.1016/s0022-0736(82)80043-5

[emmm201911115-bib-0051] Rusconi F , Ceriotti P , Miragoli M , Carullo P , Salvarani N , Rocchetti M , Di Pasquale E , Rossi S , Tessari M , Caprari S *et al* (2016) Peptidomimetic targeting of Cavbeta2 overcomes dysregulation of the L‐type calcium channel density and recovers cardiac function. Circulation 134: 534–546 27486162 10.1161/CIRCULATIONAHA.116.021347

[emmm201911115-bib-0052] Sadeghi A , Doyle AD , Johnson BD (2002) Regulation of the cardiac L‐type Ca^2+^ channel by the actin‐binding proteins alpha‐actinin and dystrophin. Am J Physiol Cell Physiol 282: C1502–C1511 11997265 10.1152/ajpcell.00435.2001

[emmm201911115-bib-0053] Shimizu W , Ohe T , Kurita T , Takaki H , Aihara N , Kamakura S , Matsuhisa M , Shimomura K (1991) Early afterdepolarizations induced by isoproterenol in patients with congenital long QT syndrome. Circulation 84: 1915–1923 1657447 10.1161/01.cir.84.5.1915

[emmm201911115-bib-0054] Stoehr A , Neuber C , Baldauf C , Vollert I , Friedrich FW , Flenner F , Carrier L , Eder A , Schaaf S , Hirt MN *et al* (2014) Automated analysis of contractile force and Ca^2+^ transients in engineered heart tissue. Am J Physiol Heart Circ Physiol 306: H1353–H1363 24585781 10.1152/ajpheart.00705.2013 PMC4116534

[emmm201911115-bib-0055] Stucker S , Kresin N , Carrier L , Friedrich FW (2017) Nebivolol desensitizes myofilaments of a hypertrophic cardiomyopathy mouse model. Front Physiol 8: 558 28824454 10.3389/fphys.2017.00558 PMC5539082

[emmm201911115-bib-0056] Takahashi K , Okita K , Nakagawa M , Yamanaka S (2007a) Induction of pluripotent stem cells from fibroblast cultures. Nat Protoc 2: 3081–3089 18079707 10.1038/nprot.2007.418

[emmm201911115-bib-0057] Takahashi K , Tanabe K , Ohnuki M , Narita M , Ichisaka T , Tomoda K , Yamanaka S (2007b) Induction of pluripotent stem cells from adult human fibroblasts by defined factors. Cell 131: 861–872 18035408 10.1016/j.cell.2007.11.019

[emmm201911115-bib-0058] Tanaka A , Yuasa S , Mearini G , Egashira T , Seki T , Kodaira M , Kusumoto D , Kuroda Y , Okata S , Suzuki T *et al* (2014) Endothelin‐1 induces myofibrillar disarray and contractile vector variability in hypertrophic cardiomyopathy‐induced pluripotent stem cell‐derived cardiomyocytes. J Am Heart Assoc 3: e001263 25389285 10.1161/JAHA.114.001263 PMC4338713

[emmm201911115-bib-0059] Tardiff JC , Carrier L , Bers DM , Poggesi C , Ferrantini C , Coppini R , Maier LS , Ashrafian H , Huke S , van der Velden J (2015) Targets for therapy in sarcomeric cardiomyopathies. Cardiovasc Res 105: 457–470 25634554 10.1093/cvr/cvv023 PMC4402369

[emmm201911115-bib-0060] Theis JL , Bos JM , Bartleson VB , Will ML , Binder J , Vatta M , Towbin JA , Gersh BJ , Ommen SR , Ackerman MJ (2006) Echocardiographic‐determined septal morphology in Z‐disc hypertrophic cardiomyopathy. Biochem Biophys Res Commun 351: 896–902 17097056 10.1016/j.bbrc.2006.10.119

[emmm201911115-bib-0061] Tseng PY , Henderson PB , Hergarden AC , Patriarchi T , Coleman AM , Lillya MW , Montagut‐Bordas C , Lee B , Hell JW , Horne MC (2017) alpha‐actinin promotes surface localization and current density of the Ca(2+) channel CaV1.2 by binding to the IQ region of the alpha1 subunit. Biochemistry 56: 3669–3681 28613835 10.1021/acs.biochem.7b00359 PMC5704914

[emmm201911115-bib-0062] Uzun AU , Mannhardt I , Breckwoldt K , Horvath A , Johannsen SS , Hansen A , Eschenhagen T , Christ T (2016) Ca(2+)‐currents in human induced pluripotent stem cell‐derived cardiomyocytes effects of two different culture conditions. Front Pharmacol 7: 300 27672365 10.3389/fphar.2016.00300 PMC5018497

[emmm201911115-bib-0063] Vandenberk B , Vandael E , Robyns T , Vandenberghe J , Garweg C , Foulon V , Ector J , Willems R (2016) Which QT correction formulae to use for QT monitoring?. J Am Heart Assoc 5: e003264 27317349 10.1161/JAHA.116.003264 PMC4937268

[emmm201911115-bib-0064] Viola HM , Hool LC (2017) The L‐type Ca(2+) channel: a mediator of hypertrophic cardiomyopathy. Channels 11: 5–7 27437722 10.1080/19336950.2016.1213053 PMC5279882

[emmm201911115-bib-0065] Walsh R , Thomson KL , Ware JS , Funke BH , Woodley J , McGuire KJ , Mazzarotto F , Blair E , Seller A , Taylor JC *et al* (2017) Reassessment of Mendelian gene pathogenicity using 7,855 cardiomyopathy cases and 60,706 reference samples. Genet Med 19: 192–203 27532257 10.1038/gim.2016.90 PMC5116235

[emmm201911115-bib-0066] Westermann D , Knollmann BC , Steendijk P , Rutschow S , Riad A , Pauschinger M , Potter JD , Schultheiss HP , Tschope C (2006) Diltiazem treatment prevents diastolic heart failure in mice with familial hypertrophic cardiomyopathy. Eur J Heart Fail 8: 115–121 16214409 10.1016/j.ejheart.2005.07.012

[emmm201911115-bib-0067] Wettwer E , Christ T , Endig S , Rozmaritsa N , Matschke K , Lynch JJ , Pourrier M , Gibson JK , Fedida D , Knaut M *et al* (2013) The new antiarrhythmic drug vernakalant: *ex vivo* study of human atrial tissue from sinus rhythm and chronic atrial fibrillation. Cardiovasc Res 98: 145–154 23341576 10.1093/cvr/cvt006

[emmm201911115-bib-0068] Wijnker PJ , Friedrich FW , Dutsch A , Reischmann S , Eder A , Mannhardt I , Mearini G , Eschenhagen T , van der Velden J , Carrier L (2016) Comparison of the effects of a truncating and a missense MYBPC3 mutation on contractile parameters of engineered heart tissue. J Mol Cell Cardiol 97: 82–92 27108529 10.1016/j.yjmcc.2016.03.003

[emmm201911115-bib-0069] Wu H , Yang H , Rhee JW , Zhang JZ , Lam CK , Sallam K , Chang ACY , Ma N , Lee J , Zhang H *et al* (2019) Modelling diastolic dysfunction in induced pluripotent stem cell‐derived cardiomyocytes from hypertrophic cardiomyopathy patients. Eur Heart J 10.1093/eurheartj/ehz326 PMC7963137 31219556

