# Supplementary material for: Disease modeling of a mutation in α‐actinin 2 guides clinical therapy in hypertrophic cardiomyopathy
Source: EMBO Mol Med. 2019 Nov 3;11(12):e11115. doi: 10.15252/emmm.201911115 (PMC6895603; doi:10.15252/emmm.201911115)
Supplement: Supplementary file 1 — Appendix [file EMMM-11-e11115-s001.pdf]

Appendix

Disease modeling of an α-actinin 2 mutation guides clinical therapy in hypertrophic cardiomyopathy

Maksymilian Prondzynski[#], Marc D. Lemoine[#], Antonia T. L. Zech, András Horváth, Vittoria Di Mauro, Jussi T. Koivumäki, Nico Kresin, Josefine Busch, Tobias Krause, Elisabeth Krämer, Saskia Schlossarek, Michael Spohn, Felix W. Friedrich, Julia Münch, Sandra D. Laufer, Charles Redwood, Alexander E. Volk, Arne Hansen, Giulia Mearini, Daniele Catalucci, Christian Meyer, Torsten Christ, Monica Patten, Thomas Eschenhagen, Lucie Carrier[*]

[#]These authors contributed equally. *Corresponding author.

Table of contents

Appendix Figure S1:	Generation of hiPSC lines and disease modeling protocol.
Appendix Figure S2:	Molecular characterization of human tissue and hiPSC-derived CMs from 2D and 3D models.
Appendix Figure S3:	Force and calcium sensitivity measurements of 3D-cultured hiPSC-derived engineered heart tissues and HCM-affected family member II.4.
Appendix Figure S4:	Validation of $Ca_V\alpha1.2$ and α-actinin 2 protein interaction by bioluminescence resonance energy transfer (BRET).
Appendix Figure S5	Representative 12-lead surface ECG
Appendix Figure S6	Fluorescent activated cell sorting (FACS) analysis of cardiac troponin T (cTnT)-positive cells.
Appendix Table S1	Analyzed HCM-associated genes.
Appendix Table S2	Fluorescent activated cell sorting (FACS) analysis of cardiac troponin T (cTnT)-positive cells.
Appendix Table S3	Sequences of PCR primers.
Appendix Table S4	Acronyms and names of genes evaluated with the nanoString nCounter® Elements technology.

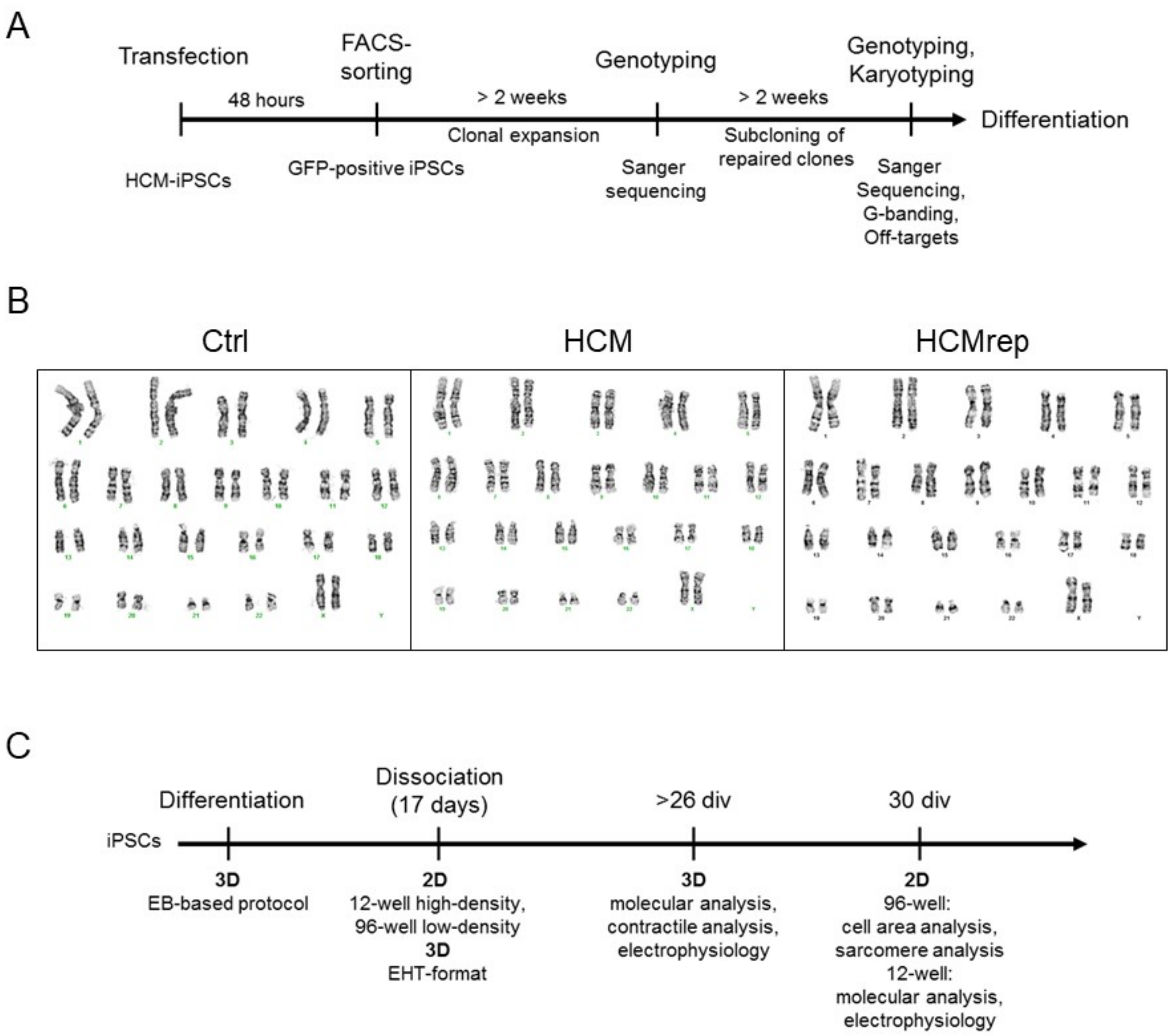

Appendix Figure S1. Generation of hiPSC lines and disease modeling protocol. (**A**) Protocol for generation of CRISPR/Cas9 isogenic control cell line HCMrep. Indicated are steps of quality assessment and approximate time needed for each step (iPSC, induced pluripotent stem cell). (**B**) G-banding results are depicted by these representative karyograms of the investigated hiPSC Ctrl- (passage 40), HCM- (passage 11) and HCMrep-line (passage 55). (**C**) Disease modeling approach for the production of hiPSC-CMs, models and experimental procedures that were used for this study (div, days *in vitro*; EB, embryoid body; EHT, engineered heart tissue).

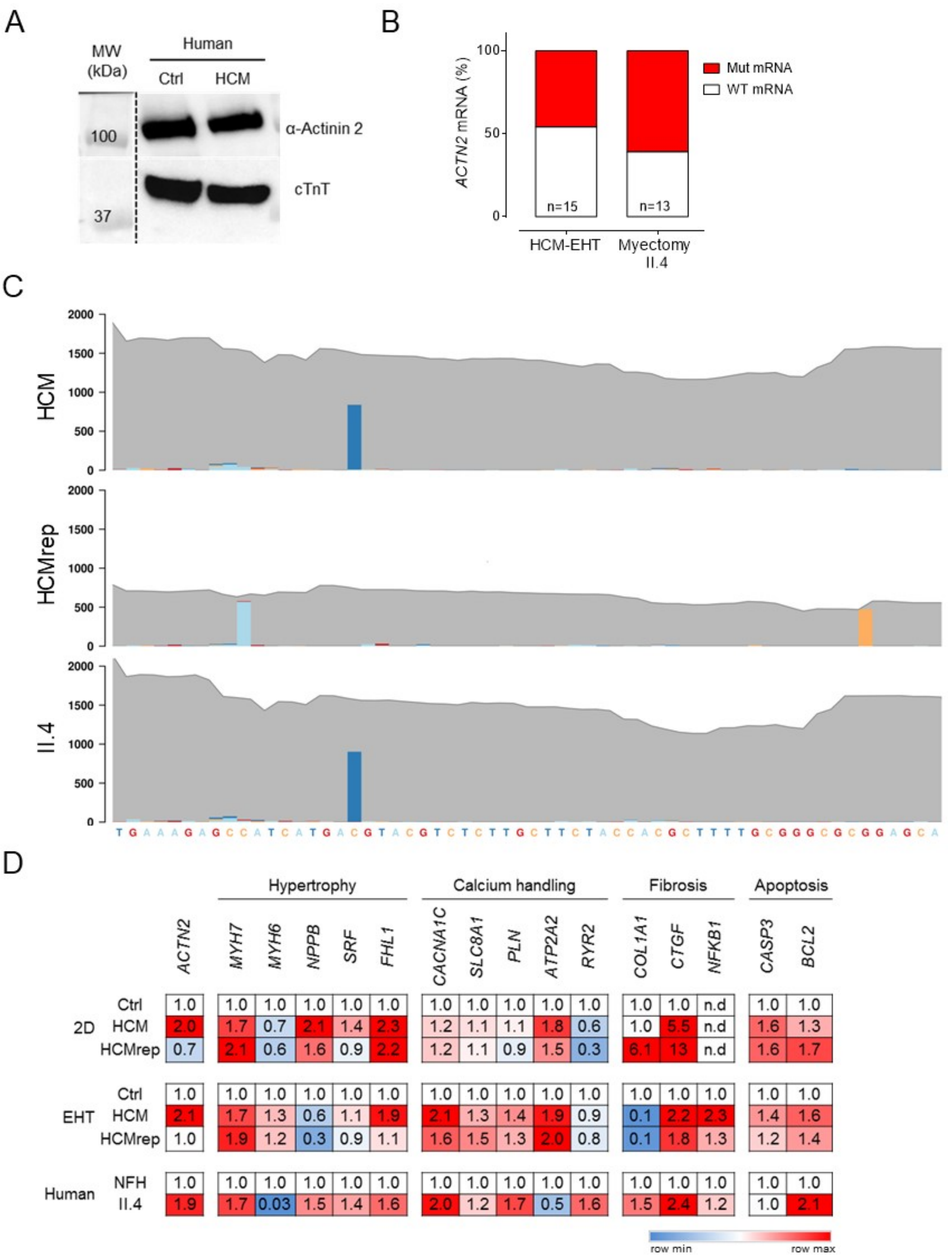

Appendix Figure S2. Molecular characterization of human tissue and hiPSC-derived CMs from 2D and 3D models. (**A**) Western blot analysis of human tissue samples stained with antibodies directed against α-actinin 2 and cardiac troponin T (cTnT; MW, molecular weight; kDa, kilodalton). (**B**) Quantification of mutant and wild-type mRNA in 3D-cultured hiPSC-derived engineered heart tissues and HCM-affected family member II.4. Transcripts were quantified by conversion of RNA to cDNA, subcloning of amplified *ACTN2* RT-PCR

fragments and validation by sequencing (n=number of picked and sequenced clones; HCM-EHTs (n=15); HCM tissue (n=13); mut, mutant; WT, wild type). Data are expressed as the % of WT versus mutant mRNA transcripts. (**C**) RNA sequencing analysis revealed mismatches in the genomic range of chromosome 1 from 236.735.660 bp to 236.735.720 bp showing heterozygous C>T mutation of base 236.735.677 in ~ 50% of analyzed reads (blue bars out of grey zone) in both HCM-EHTs (n=7/6) and patient II.4 septal myectomy. CRISPR/Cas9 engineered isogenic control HCMrep-EHTs (n=7/3) showed no C>T transition at base 236.735.677, but the simultaneously introduced homozygous silent C>A and G>C mutations at bases 236.735.668 (light blue bar) and 236.735.714 (orange bar). (**D**) Gene expression analysis in hiPSC-CMs and -EHTs, performed with the nanoString nCounter® Elements technology. Data were normalized to housekeeping genes (*ABCF1*, *CLTC*, *GAPDH*, *ACTB)* and related to Ctrl (n=number of pooled samples/differentiations; 2D: Ctrl (n=16/3); HCM (n=15/3); HCMrep (n=18/3); EHTs: Ctrl (n=7/4); HCM (n=7/4); HCMrep (n=7/3)). Human samples were normalized to housekeeping genes (see method) and related to Ctrl (n=pool of 9 non-failing heart (NFH) tissues and n=1 for septal myectomy of patient II.4).

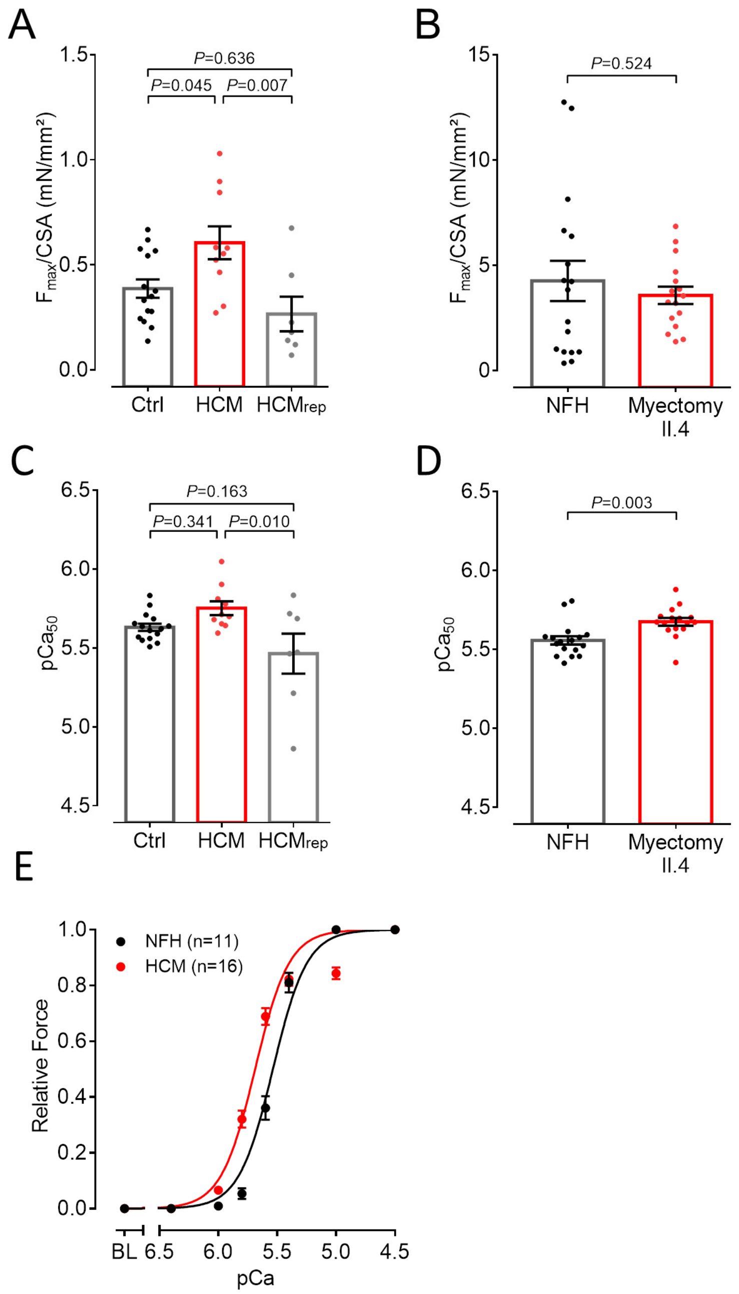

Appendix Figure S3. Force and calcium sensitivity measurements of 3D-cultured hiPSC-derived engineered heart tissues and HCM-affected family member II.4. (**A**) Functional parameters of maximal force (F_{max}) normalized to cross-sectional area (CSA) obtained from permeabilized EHT-strips (n=number of analyzed muscle strips; Ctrl (n=15), HCM (n=10), HCMrep (n=7)). Data are expressed as mean±SEM, one-way ANOVA plus Bonferroni's post-test. (**B**) Functional parameters of force obtained from muscle strips of non-failing human heart (NFH; n=15) and septal myectomy of index patient II.4 (n=16). Data are expressed as mean±SEM. Unpaired Student's *t*-test. (**C**) pCa_{50} values of EHT muscle strips from Ctrl (n=15), HCM (n=10), HCMrep (n=7). Data are expressed as mean±SEM, one-way ANOVA with Bonferroni´s post-test. (**D**) pCa_{50} values of non-failing heart (NFH) tissue (n=15), septal myectomy of index patient II.4 (n=16). Data are expressed as mean±SEM, unpaired Student's *t*-test. (**E**) pCa_{50} curve of non-failing heart (NFH) tissue (n=11), septal myectomy from patient II.4 (n=16)). Data are expressed as mean±SEM. Concentration response curves were fitted to the data points and force-pCa relationship comparison was done by using extra sum-of-squares F-test.

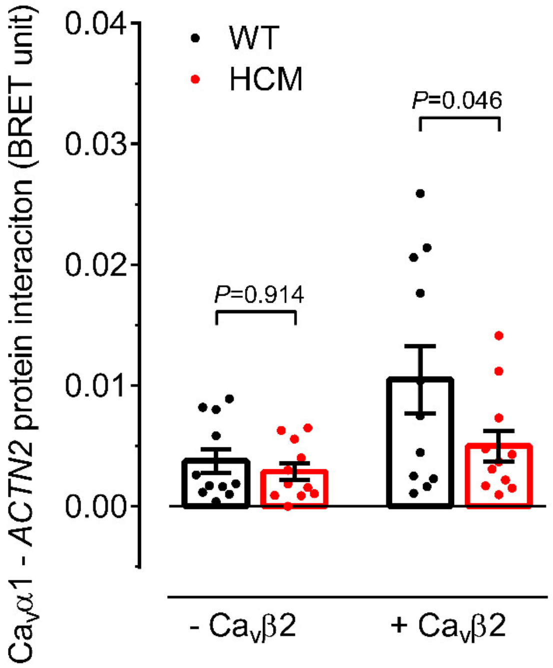

Appendix Figure S4. Validation of Ca$_v$α1.2 and α-actinin 2 protein interaction by bioluminescence resonance energy transfer (BRET) assay. HL-1 transfected cells with α-actinin 2 wildtype (WT, n=11) and mutant (HCM, n=11) proteins were analyzed for their interaction with the Ca$_v$α1.2 and Ca$_v$β2 domain. Data are expressed as mean±SEM, two-way ANOVA, followed by Sidak's multiple comparisons test.

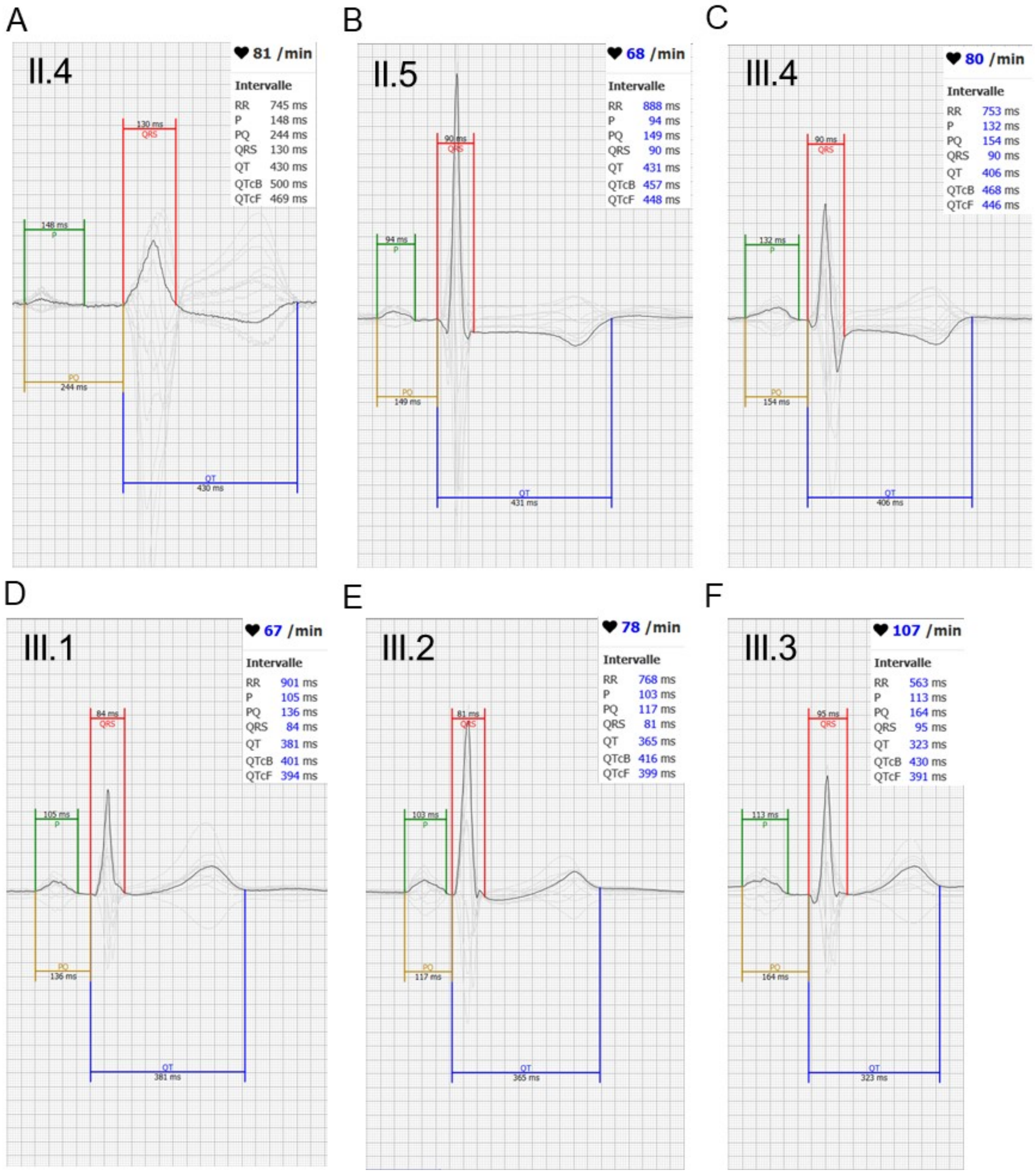

Appendix Figure S5. Representative 12-lead surface ECG with averaged signal and overlay of all leads (in grey, apart from V5 in black) of the genotyped family sorted by positive *ACTN2* (**A**, patient II.4; **B**, patient II.5; **C**, patient III.4) and negative (**D**, patient III.1; **E**, patient III.2; **F**, patient III.3).

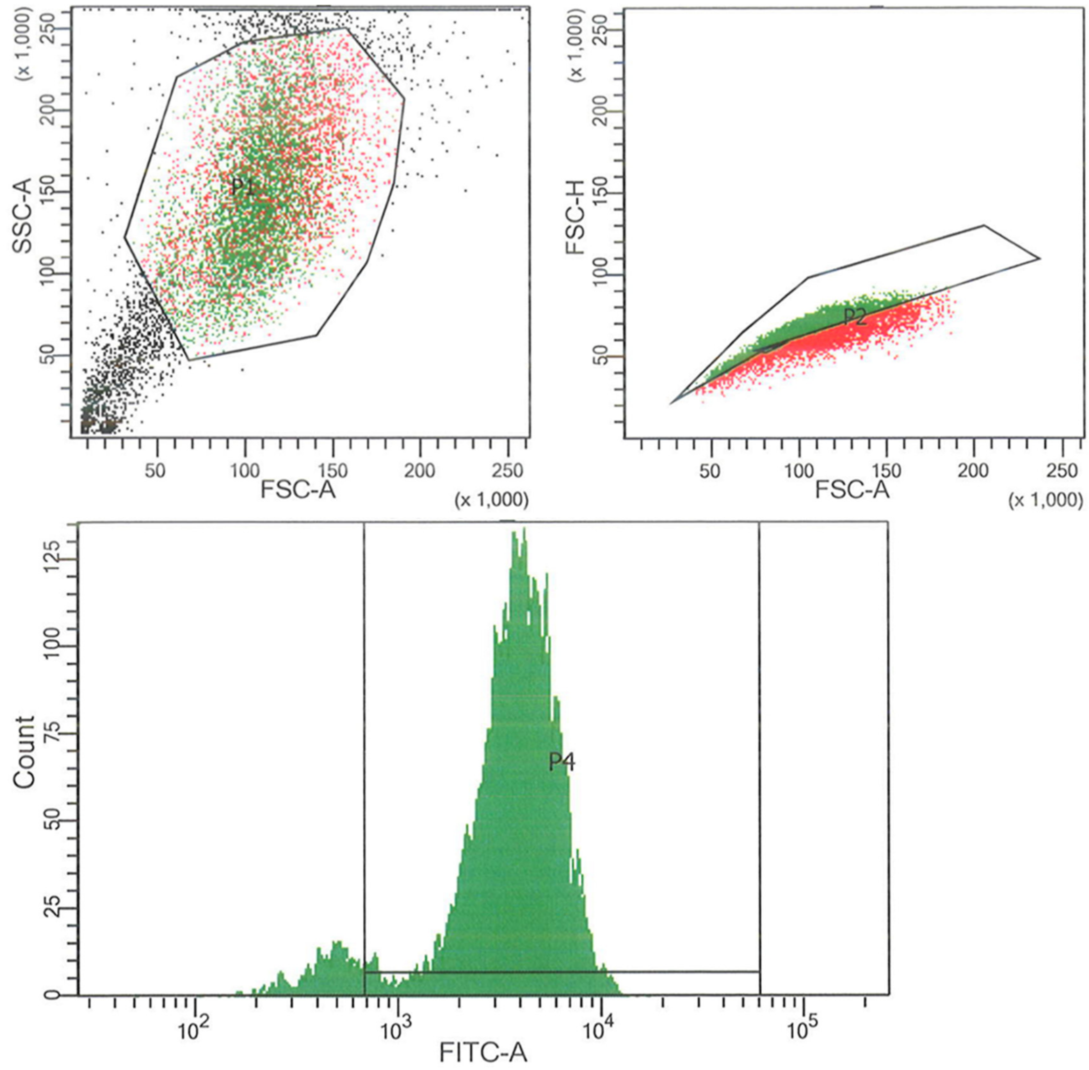

Population	#Events	%Parent	%Total
All Events	10,000	####	100.0
P1	7,105	71.0	71.0
P2	3,974	55.9	39.7
P4	3,684	92.7	36.8

Appendix Figure S6. Fluorescent activated cell sorting (FACS) analysis of cardiac troponin T (cTnT)-positive cells. Representative FACS-plots showing gating strategy for evaluation of cTnT-positive cells after cardiac differentiation. Fixed HCM-cardiomyocytes were stained with a directly labeled FITC cTnT antibody, quantified with the BD FACSCANTO II (BD Biosciences) and analyzed with the FACSDiva software (BD Biosciences).

Appendix Table S1. Analyzed HCM-associated genes

Gene	RefSeq Accession
ACTC1	NM_005159.4
ACTN2	NM_001103.2
ANKRD1	NM_014391.2
CSRP3	NM_003476.3
FHL1	NM_001159702.2; NM_001449.4
FLNC	NM_001458.4
GLA	NM_000169.2
LAMP2	NM_002294.2; NM_001122606.1; NM_013995.2
MYBPC3	NM_000256.3
MYH7	NM_000257.2
MYL2	NM_000432.3
MYL3	NM_000258.2
PLN	NM_002667.3
PRKAG2	NM_016203.3
TNNC1	NM_003280.2
TNNI3	NM_000363.4
TNNT2	NM_001276345.1
TPM1	NM_001018005.1 AY640414.1
TTR	NM_000371.3

Appendix Table S2. Analyzed off-targets in HCMrep by PCR.

NCBI accession number		off-target position
NG_029480	Exon	g.81,841-81,860
NM_016642	Exon	c.1037-1056
NG_013304	Exon	g.14,262-14,281
NG_029938	Exon	g.149,593-149,612
NM_000827	Exon	c.2942-2961
NG_009061	Exon	g.24,969-24,988
NM_025268	Exon	c.898-917
NC_018924	Intron	g.109,195,197-109,195,219
NC_018930	Intron	g.39,202,757-39,202,779
NC_018931	Intron	g.60,206,273-60,206,295
NC_018919	Intron	g.81,458,539-81,458,561
NC_018916	Intron	g.16,615,937-16,615,959
NC_018929	Intron	g.73,770,186-73,770,208

Appendix Table S3. Sequences of PCR primers

Primers	Forward (5´ to 3´)	Reverse (5´ to 3´)
ACTN2 genotyping	ggcccatgaaacacagaaat	agggccattcttcctcaagg
NG_029480	ggggtgtatggtgttcttgg	ggcaggaggacatggtttg
NM_016642	cttttgctttcctggtggct	gtctcctctggacagtctgc
NG_013304	ggaagagaagacactgggct	gactgagtgtgtgcagctgg
NG_029938	ccaaaggttcagagaagggc	cccggaagatgatggtgtct
NM_000827	tttgtttgatcccacagcaa	ggtctccatctgctccagtt
NG_009061	agggtgcttgagttgatcct	tgttggtggcagtggaca
NM_025268	ccgcagaagatgatgctgta	ggctgcagctccagtgatag
NC_018924	ttctgggttcaagccatcct	aagctcactgaaaggaaaggt
NC_018930	cttccagtccagagcaagtg	cagtcaaatcccagctctgc
NC_018931	tcagtttctacggccactgt	tgaaacctctctcttgccgt
NC_018919	agtgggttgctgcagagtaa	acaggtgtgagccatgtacc
NC_018916	tctgcactgtgtaggtcatgt	tgatgagaaaacgggaggca
NC_018929	gcaaggcatccacgaatagt	gctatttggggcactttggt
Allele-specific mRNA analysis	gccatggaaatcgctgagaa	atcctgttagccgctgtctc

Appendix Table S4. Acronyms and names of genes evaluated with the nanoString nCounter® Elements technology.

Gene	Acronym	Accession number (NCBI)
Alpha-Actinin 2	*ACTN2*	NM_001103.2
Myosin heavy chain 7	*MYH7*	NM_000257.2
Myosin heavy chain 6	*MYH6*	NM_002471.3
Natriuretic peptide B	*NPPB*	NM_002521.2
Serum response factor	*SRF*	NM_003131.3
Four-and-a-half-LIM-domains 1	*FHL1*	NM_001449.4
L-Type Ca^{2+} channel	*CACNA1C*	NM_199460.2
Natrium-Calcium Exchanger NCX	*SLC8A1*	NM_021097.1
Phospholamban	*PLN*	NM_002667.3
ATPase sarcoplasmic/endoplasmic reticulum Ca^{2+} transporting 2	*ATP2A2*	NM_001681.3
Ryanodine receptor 2	*RYR2*	NM_001035.2
Collagen type I alpha 1	*COL1A1*	NM_000088.3
Connective tissue growth factor	*CTGF*	NM_001901.2
Nuclear factor kappa B subunit 1	*NFKB1*	NM_003998.2
Caspase 3	*CASP3*	NM_032991.2
BCL2, apoptosis regulator	*BCL2*	NM_000657.2
ATP Binding Cassette Subfamily F Member 1	*ABCF1*	NM_001090.2
Clathrin Heavy Chain	*CLTC*	NM_004859.2
Glyceraldehyde 3-phosphate dehydrogenase	*GAPDH*	NM_002046.3
Beta-actin	*ACTB*	NM_001101.2
Phosphoglycerate kinase 1	*PGK1*	NM_000291.2
Tubulin Beta Class I	*TUBB*	NM_178014.3
