# Supplementary material for: Disease modeling of a mutation in α‐actinin 2 guides clinical therapy in hypertrophic cardiomyopathy
Source: EMBO Mol Med. 2019 Nov 3;11(12):e11115. doi: 10.15252/emmm.201911115 (PMC6895603; doi:10.15252/emmm.201911115)
Supplement: Supplementary file 8 — Source Data for Figure 1 [file EMMM-11-e11115-s006.pdf]

a

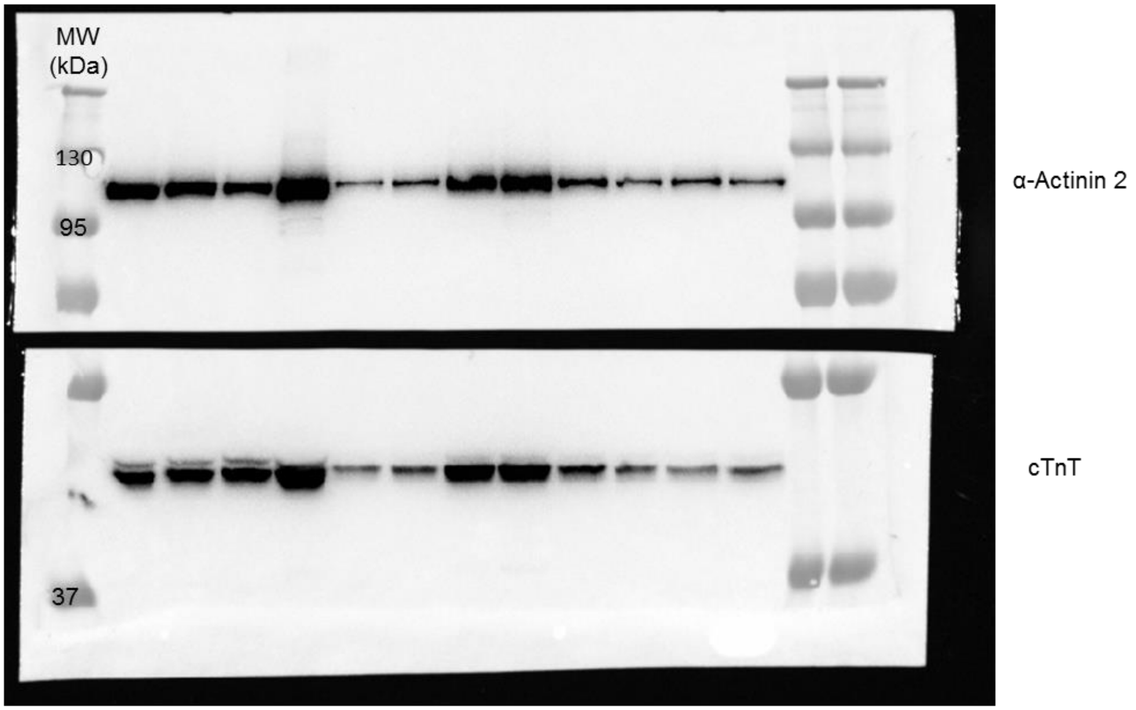

b

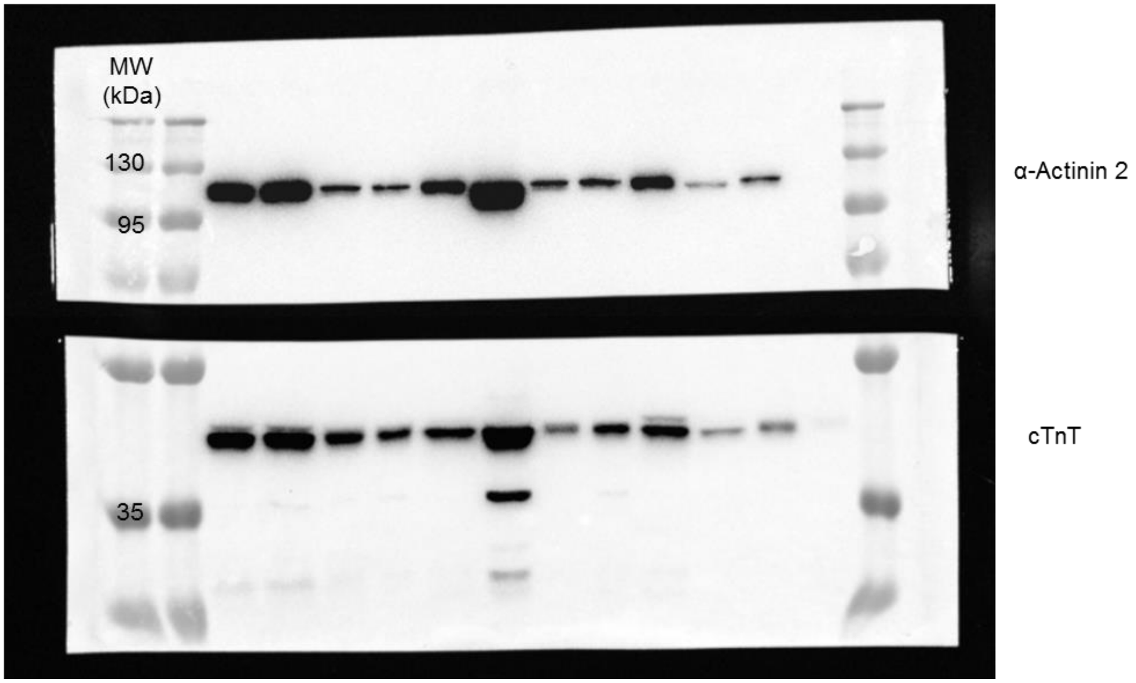

Source data to Figure 1: (a) Full western blot to the corresponding Figure 1C. (b) Full western blot to the corresponding Figure 1D.
