# Supplementary material for: Disease modeling of a mutation in α‐actinin 2 guides clinical therapy in hypertrophic cardiomyopathy
Source: EMBO Mol Med. 2019 Nov 3;11(12):e11115. doi: 10.15252/emmm.201911115 (PMC6895603; doi:10.15252/emmm.201911115)
Supplement: Supplementary file 6 — Source Data for Appendix [file EMMM-11-e11115-s007.pdf]

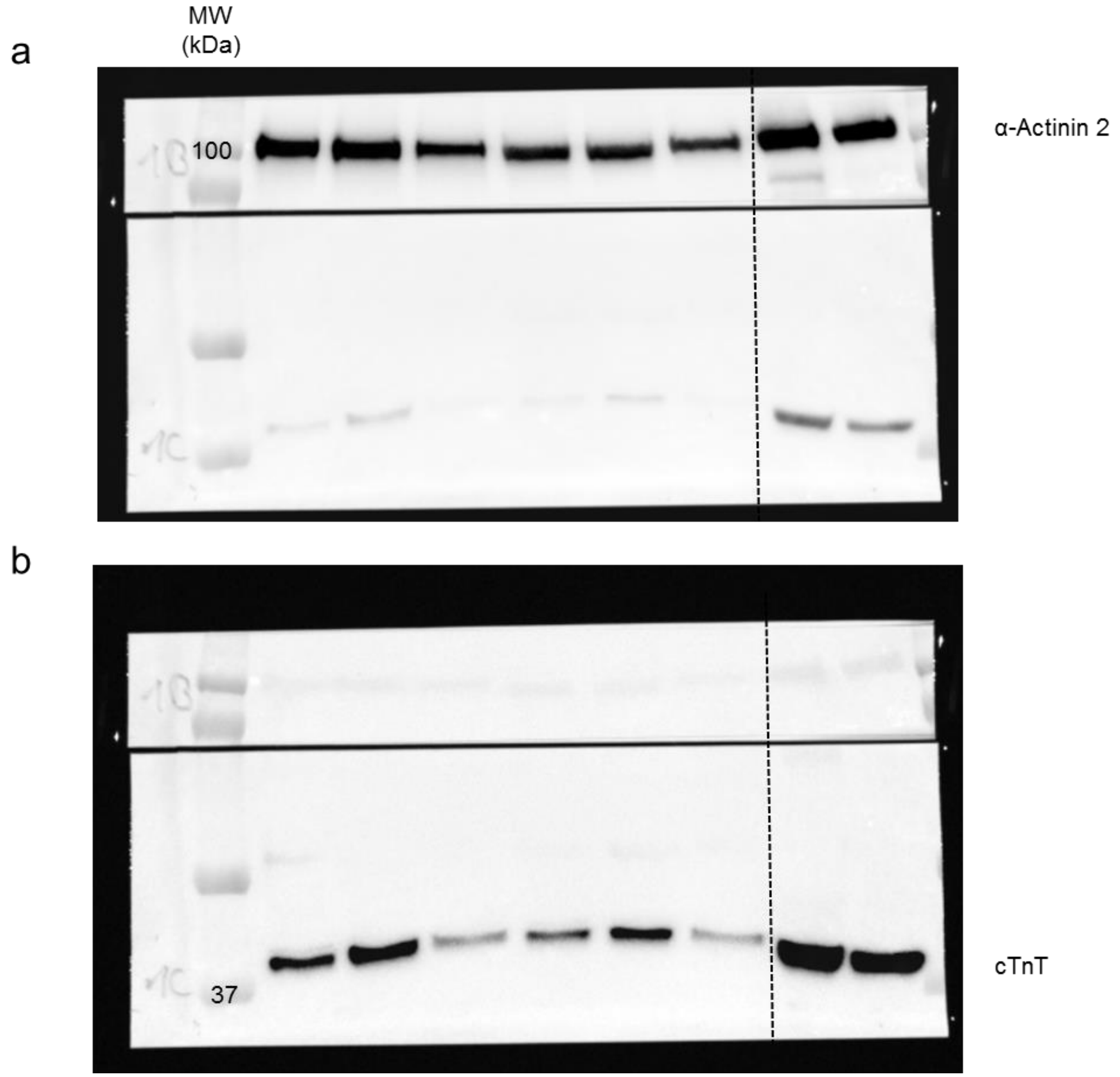

Source data to Appendix Figure S2: (a) Full western blot to the corresponding Appendix Fig S2A showing the detection of α-actinin 2 (b) Full western blot to the corresponding Appendix Fig S2A showing the detection of cardiac troponin T (cTnT). The dashed line corresponds to the depiction of the western blot in Supplementary Figure 2b, since additional samples on this blot were not directly related to this study and therefore not included in the analysis.
